# CCR6 Deficiency Impairs IgA Production and Dysregulates Antimicrobial Peptide Production, Altering the Intestinal Flora

**DOI:** 10.3389/fimmu.2017.00805

**Published:** 2017-07-11

**Authors:** Ya-Lin Lin, Peng-Peng Ip, Fang Liao

**Affiliations:** ^1^Taiwan International Graduate Program in Molecular Medicine, National Yang-Ming University and Academia Sinica, Taipei, Taiwan; ^2^Institute of Biomedical Sciences, Academia Sinica, Taipei, Taiwan

**Keywords:** CCR6, IgA, Peyer’s patch, antimicrobial peptide, isolated lymphoid follicle

## Abstract

Intestinal immunity exists as a complex relationship among immune cells, epithelial cells, and microbiota. CCR6 and its ligand–CCL20 are highly expressed in intestinal mucosal tissues, such as Peyer’s patches (PPs) and isolated lymphoid follicles (ILFs). In this study, we investigated the role of the CCR6–CCL20 axis in intestinal immunity under homeostatic conditions. CCR6 deficiency intrinsically affects germinal center reactions in PPs, leading to impairments in IgA class switching, IgA affinity, and IgA memory B cell production and positioning in PPs, suggesting an important role for CCR6 in T-cell-dependent IgA generation. CCR6 deficiency impairs the maturation of ILFs. In these follicles, group 3 innate lymphoid cells are important components and a major source of IL-22, which stimulates intestinal epithelial cells (IECs) to produce antimicrobial peptides (AMPs). We found that CCR6 deficiency reduces IL-22 production, likely due to diminished numbers of group 3 innate lymphoid cells within small-sized ILFs. The reduced IL-22 levels subsequently decrease the production of AMPs, suggesting a critical role for CCR6 in innate intestinal immunity. Finally, we found that CCR6 deficiency impairs the production of IgA and AMPs, leading to increased levels of *Alcaligenes* in PPs, and segmented filamentous bacteria in IECs. Thus, the CCR6–CCL20 axis plays a crucial role in maintaining intestinal symbiosis by limiting the overgrowth of mucosa-associated commensal bacteria.

## Introduction

The mammalian intestine harbors trillions of commensal bacteria, which left unchecked, would invade host tissues. Both innate and adaptive immune cells in intestinal tissues are important for limiting tissue invasion by the resident commensal bacteria (1, 2), and maintenance of the symbiotic relationship between the bacteria and host is a critical factor in intestinal homeostasis. IgA and antimicrobial peptides (AMPs) serve as the first line of the mucosal defense system. IgA binds and neutralizes intestinal microbes and antigens (3, 4), while AMPs exert bactericidal effects and spatially constrain intestinal bacteria (5, 6). Intestinal IgA is produced mainly from Peyer’s patches (PPs) in a T-cell-dependent manner (7–9). Activated PP B cells form germinal centers (GC), where B cells undergo intensive proliferation, class-switch recombination, and somatic hypermutation, and eventually differentiate into IgA memory B cells or IgA plasmablasts, which are capable of producing high-affinity T-cell-dependent IgA (TD-IgA) (10). AMPs, the other important front-line defense system for intestinal barriers, are secreted by enterocytes and Paneth cells in intestines (11–13). The secretion of AMPs by intestinal epithelial cells (IECs) can be induced by microbiota (5) or stimulated by group 3 innate lymphoid cell (ILC3)-derived IL-22 (14). Lymphoid tissue inducer (LTi) cells express the transcription factor RORγt and are a subset of ILC3s (15–18). These LTi–ILC3s are not only an important source of IL-22 (19) but they are also essential to respond to microbiota stimulation by initiating the formation of PPs at the embryonic stage and isolated lymphoid follicles (ILFs) at postnatal stages (16, 20–22). Thus, the LTi–ILC3s play crucial roles in both innate and adaptive immunity to maintain intestinal homeostasis.

CCR6 is expressed in naive and memory B cells (23, 24), memory T cells (25), Th17 cells (26), T_Foxp3_ cells (27), immature dendritic cells (DCs) (28), monocytes (29), and subsets of ILC3s (18). CCL20, the sole chemokine ligand of CCR6 (30, 31), is constitutively expressed in gut tissues and abundantly expressed in the follicle-associated epithelium (FAE) in PPs (32, 33), while CCR6 is highly expressed in the subepithelial dome (SED) of PPs (33). Based on the high expression of CCR6 and CCL20 in PPs, one may speculate that the CCR6–CCL20 axis plays a critical role in gut immunity (34), particularly in humoral immunity, since PPs are the major sites for IgA induction (7). Using CCR6^−/−^ mice, researchers have shown that the CCR6–CCL20 axis is important for protecting against mucosal infectious pathogens, such as rotavirus (32), *S*. *typhimurium* (35), and respiratory syncytial virus (36). On the contrary, CCR6^−/−^ mice are resistant to oral infection of *Yersinia enterocolitica* (37). In addition, the CCR6–CCL20 axis plays a role in human intestinal inflammatory diseases. CCL20 expression is elevated in patients with inflammatory bowel disease (38), and the gene was identified as a susceptibility factor for Crohn’s disease (39) by genome-wide association studies. Thus, it is clear that the CCR6–CCL20 axis is involved in intestinal infection and inflammation, but its specific roles in maintaining intestinal homeostasis are not fully described.

To gain a basic understanding of how the CCR6–CCL20 axis affects intestinal immunity, we examined intestinal tissues under steady-state conditions in CCR6^−/−^ mice. To ensure comparable genetic backgrounds and environments, we used littermate CCR6^+/+^ (WT) and CCR6^−/−^ mice under the same housing conditions. We examined the effects of CCR6 on the production of IgA and AMPs, the most important molecules regulating intestinal adaptive and innate immunity, respectively. Our study shows that CCR6 deficiency attenuates the production of TD-IgA and interferes with the maintenance of IgA-bearing memory B cells. Of note, we found that CCR6 is dispensable for B cell migration toward PPs. In addition, we found that CCR6 deficiency affects the innate response mediated by ILC3–LTi cells. These cells are a major source of IL-22, which stimulates IECs to produce AMPs. Our study highlights important roles for the CCR6–CCL20 axis in both innate (AMP producing) and adaptive immunity (IgA producing) in maintaining intestinal homeostasis.

## Materials and Methods

### Mice

CCR6^−/−^ mice were generated as described (40) and kindly provided by Dr. J. Farber (Laboratory of Molecular Immunology, National Institute of Allergy and Infectious Diseases, National Institutes of Health, Bethesda, MD, USA). CCR6^−/−^, WT, CD45.1, Rag1^−/−^, and JH^−/−^ mice on the C57BL/6 background were housed in specific pathogen-free conditions at the Institute of Biomedical Sciences, Academia Sinica (Taipei, Taiwan). CD45.1^+/+^CCR6^+/−^ heterozygous breeders were generated by crossing CCR6^+/−^ to CD45.1 mice. To minimize variations in experimental outcomes that may be caused by genetic background or environmental factors, we performed all experiments with CCR6^+/+^ and CCR6^−/−^ littermates that were generated from heterozygous breeders (CCR6^+/−^ × CCR6^+/−^). Age-matched littermates (8–12 weeks) were used in the study and housed under the same conditions. All animal experiments were approved by the Institutional Animal Care and Utilization Committee at Academia Sinica and performed in accordance with institutional guidelines.

### Isolation of Lymphocytes from PPs and Intestinal Lamina Propria

Peyer’s patches were excised, placed in RPMI 1640 medium (Gibco) containing 10% FBS (Hyclone), 100 U/ml penicillin, 100 µg/ml streptomycin, and 10 mM HEPES (Gibco), and mechanically dissociated by gently tearing the tissues with 27 G needles. Tissue debris was removed by filtering cell suspensions through a 40-µm cell strainer. Lamina propria lymphocytes were isolated following the method described by Reissig et al. with modifications (41). In brief, intestines were cut longitudinally, washed in PBS to remove intestinal contents, and cut into small pieces with 0.5 cm in length. The pieces of intestine were incubated with predigestion buffer containing HBSS without Ca^2+^ Mg^2+^, 5% FBS, 10 mM HEPES, 1 mM DTT, and 5 mM EDTA with gentle shaking for 15 min at 37°C to remove epithelium. After incubation with predigestion buffer two times, the pieces of intestine were incubated with wash buffer (HBSS without Ca^2+^ Mg^2+^ containing 5% FBS and 10 mM HEPES) with gentle shaking for 15 min at 37°C. Intestine pieces were further cut into 1-mm pieces and incubated with digestion buffer containing HBSS with Ca^2+^ Mg^2+^, 10% FBS, 10 mM HEPES, 0.5 mg/ml type IV collagenase (Sigma-Aldrich), and 200 U/ml DNase I (Bioshop). After 30-min incubation at 37°C, cells were collected and subjected to Percoll (GE Healthcare) gradient centrifugation (40 and 80%). Cells at the interface were collected, washed twice with wash buffer, and resuspended in RPMI medium containing 10% FBS.

### Isolation of Naive B Cells from PPs and Adoptive Transfer Experiments

Peyer’s patch lymphocytes were isolated and stained with FITC-conjugated anti-mouse IgD (clone 11-26) (BioLegend) followed by incubation with anti-FITC magnetic beads (Miltenyi). Naive B cells were obtained by the purification of IgD-positive cells, by positive selection using MACS columns (Miltenyi). The purity was >98%. For adoptive transfer experiments, 2 × 10^6^ of PP naive B cells were retro-orbitally transferred into Rag1^−/−^ or JH^−/−^ mice. Recipient mice were sacrificed 2 weeks post-transfer, and IgA production was analyzed.

### Bone Marrow Chimeras

Six-week-old Rag1^−/−^ mice were irradiated with sublethal dose (4 Gy) of X-ray followed by intraperitoneal injection with 1 × 10^7^ mixed bone marrow cells isolated from WT and CCR6^−/−^ mice with different isoforms of CD45 (WT:CCR6^−/−^ = 1:1). For the bone marrow chimera experiments, 15 chimeric mice were generated: 5 mice were reconstituted with mixed bone marrow from WT (CD45.1^+^CD45.2^+^) and CCR6^−/−^ (CD45.1^+/+^); 4 mice were reconstituted with mixed bone marrow from WT (CD45.1^+/+^) and CCR6^−/−^ (CD45.1^+^CD45.2^+^); 6 mice were reconstituted with mixed bone marrow from WT (CD45.1^+/+^) and CCR6^−/−^ (CD45.2^+/+^). In these experiments, we used CD45 congenic markers (CD45.1^+/+^, CD45.2^+/+^, CD45.1^+^CD45.2^+^) in each genotype in order to minimize variations that might be caused by the use of different congenic markers. After the bone marrow was reconstituted for at least 3 months, chimeric mice were used in experiments.

### Antibodies for Flow Cytometry

The following fluorochrome-conjugated antibodies used in this study were purchased from eBioscience: anti-mouse CD45 (clone 30-F11), anti-mouse CD3e (clone 145-2C11), anti-mouse CD4 (clone RM4-5), anti-mouse lineage (clones 11A2, RB6-8C5, M1/70, RA3-6B2, TER-199), anti-mouse CD95 (clone 15A7), anti-mouse CD38 (clone 90), anti-mouse IgD (clone 11-26), anti-mouse IgM (clone II/41), anti-mouse CD73 (clone TY/11.8), anti-mouse PD-L2 (clone TY25), and anti-mouse RORγt (clone B2D). The following fluorochrome-conjugated antibodies were purchased from BioLegend: anti-mouse CD45.1 (clone A20), anti-mouse CD45.2 (clone 104), anti-mouse I-A^b^ (clone AF6-120.1), anti-mouse PD-1 (clone 29F.1A12), anti-mouse CCR6 (clone 29-2L17), anti-mouse CD127 (clone A7R34), anti-mouse CD117 (clone 2B8), anti-mouse Foxp3 (clone MF-14), and anti-mouse IL-17 (clone TC11-18H10.1). Texas-red-conjugated anti-mouse IgA (goat polyclonal) was purchased from SouthernBiotech, and fluorochrome-conjugated anti-mouse IgG1 (clone X56), anti-mouse B220 (clone RA3-6B2), and anti-mouse CXCR5 (clone 2G8) were purchased from BD Biosciences.

### Flow Cytometry Analysis

For surface staining, cells were washed with FACS staining buffer (HBSS with Ca^2+^ Mg^2+^, 1% FBS, 10 mM HEPES, and 0.1% NaN_3_) followed by incubation with Fc blocker (anti-CD16/32, clone 2.4G2, BD Biosciences) on ice for 15 min and stained with antibodies against surface markers on ice for 30 min. After two washes with PBS, cells were stained with fixable viability dye (eBioscience) on ice for 10 min. After two washes with PBS, cells were fixed in PBS containing 0.8% paraformaldehyde followed by FACS analysis. For intracellular staining of transcription factors, cells were first stained with surface markers followed by fixation and intracellular staining using Foxp3/Transcription factor staining buffer (eBioscience) according to the manufacturer’s instruction. For intracellular cytokine staining, cells were stimulated with 20 µg/ml PMA (Sigma-Aldrich) and 1 µM ionomycin (Sigma-Aldrich) in the presence of 5 µg/ml brefeldin A (BioLegend) for 4 h at 37°C prior to staining with surface markers and viability dye. After surface staining, cells were fixed with 2% paraformaldehyde, washed and permeabilized with intracellular staining (IC) buffer (1% BSA and 0.1% saponin in PBS), blocked with 2% normal rat serum in IC buffer for 15 min at room temperature followed by staining with fluorochrome-conjugated antibodies to cytokines for 30 min at room temperature. Cells were washed with IC buffer twice, resuspended in FACS staining buffer, and subjected to FACS analysis. FACS analyses were performed using LSRII system (BD Biosciences), and data were analyzed using FlowJo software (TreeStar). The fold change of mean fluorescence intensity (MFI) was presented as MFI from samples divided by the MFI of a given WT mouse in each experiment. Gating strategies for the FACS analysis are depicted in Figures S2–S6 in Supplementary Material.

### Apoptosis Analysis

Active caspase 3 was detected using CaspGLOW™ Fluorescein Active Caspase-3 Staining Kit (eBioscience) according to the manufacturer’s instructions. Cells were incubated with FITC-DEVD-FMK or Z-VAD-FMK for 30 min at 37°C prior to surface staining. The cells were then fixed with 0.8% paraformaldehyde in PBS followed by FACS analysis. For the detection of Annexin V, cells were stained with surface markers, washed with binding buffer (10 mM HEPES, 140 mM NaCl, 2.5 mM CaCl_2_ in PBS), and incubated with Alexa Fluor 647-conjugated Annexin V (Invitrogen) for 10 min at room temperature. After two washes with binding buffer, cells were fixed with 0.8% paraformaldehyde in PBS and subjected to FACS analysis.

### Analysis of Fecal Bacteria by Flow Cytometry

IgA coated on fecal bacteria was examined following the methods described by Kawamoto et al. with modifications (42). Feces were collected from mouse cecum, weighted, and homogenized in protease inhibitor solution (0.01% NaN_3_, 20 mM EDTA, and protease inhibitor 1:1,000 v/v in PBS) at 100 mg/ml. Homogenates were centrifuged at 400 × *g* for 5 min at 4°C to remove large particles. Supernatants were collected and filtered through a 40-µm cell strainer. Fecal bacteria were obtained by further centrifugation at 10,000 × *g* at 4°C for 5 min. Bacteria-free supernatants were collected and subjected to ELISA analysis of IgA. After removal of bacteria-free supernatants, fecal bacteria were resuspended in staining buffer (1% BSA in PBS) and 100 µl of bacteria suspension were used for IgA staining. To detect IgA coated on fecal bacteria, fecal bacteria were washed with staining buffer once, blocked with 5% normal goat serum for 1 h at 4°C, and incubated with biotin-conjugated goat anti-mouse IgA (SouthernBiotech) for 20 min at 4°C followed by incubating with Alexa Fluor 647-conjugated Streptavidin (Invitrogen) for 20 min at 4°C. After three washes with staining buffer, bacteria were resuspended in staining buffer containing DAPI (1 µg/ml) and subjected to FACS analysis with LSRII (BD Biosciences). Bacteria were gated on DAPI positive events (Figure S3 in Supplementary Material).

### ELISA

For the analysis of antibody concentrations in sera or feces, 96-well microtiter plates were coated with 2 µg/ml of goat anti-mouse antibodies (H + L) (SouthernBiotech) in 50 mM carbonate/bicarbonate coating buffer at 4°C overnight. Plates were washed several times with PBST (0.05% Tween 20 in PBS) and blocked with 1% BSA in PBS for 2 h at room temperature. Serially diluted sera or fecal bacteria-free supernatants were added into the plates and incubated overnight at 4°C. Serial diluted recombinant mouse IgA (SouthernBiotech), IgM, IgG1, IgG2a, and IgG2b (BioLegend) were used as standard. After overnight incubation, the plates were washed with PBST, and HRP-conjugated goat anti-mouse IgA, IgM, IgG1, IgG2a, and IgG2b (SouthernBiotech) were added into the plates followed by 1-h incubation at RT. The plates were then washed with TBST followed by adding 3,3′,5,5′-tetramethylbenzidine substrate (KPL) for color development. The enzymatic reaction was stopped by adding 1 M H_2_SO_4_, and the plates were subjected to the measurement of OD 450 nm. For the detection of CXCL13 by ELISA, mice PPs were weighed and homogenized in protease inhibitor solution (0.01% NaN_3_, 20 mM EDTA, and protease inhibitor 1:1,000 v/v in PBS) at 50 mg/ml. Tissue debris was removed by centrifugation with 1,000 × *g* at 4°C. Total tissue proteins were determined by Bradford protein assay (Bio-Rad Laboratories), and CXCL13 were measured by ELISA according to the manufacturer’s instructions (R&D systems). The concentrations of chemokines in PPs were normalized to micrograms of tissue proteins.

### Gene Expression by Quantitative PCR Analysis

Mouse intestines were equally divided into three parts and PPs were removed. The distal part of the intestine (ileum) was cut longitudinally, followed by washing with PBS, and the epithelia were collected by gently scrapping the inner layer of intestines with glass slides. The scrapped epithelia were referred to ileum scrapes in this study. The remaining intestines containing crypts were referred to ileum crypts. Total RNA was extracted using Trizol reagent (Invitrogen) according to the manufacturer’s instructions. Total RNA was treated with RQ1 RNase-free DNase (Promega), and the first strand of cDNA was synthesized by using random hexamers and SuperScript III Reverse Transcriptase (Invitrogen). Quantitative PCR was performed on ABI prism 7500 (Applied Biosystems) using SYBR Green Master mix (Thermo Scientific) or TaqMan probes (Applied Biosystems). The thermal cycling protocol was 1 cycle at 50°C for 2 min and 95°C for 10 min, followed by 40 cycles of 95°C for 15 s and 60°C for 1 min. The resultant PCR products were measured using an ABI prism 7500 Sequence Detection System (Applied Biosystems). Gene expression was normalized to GAPDH and analyzed using the ΔΔ*C*_t_ method. DNA sequences of the primer pairs used in this study are listed in Table S1A in Supplementary Material, and Taqman probes are listed in Table S1B in Supplementary Material.

### Microbiota Analysis

Feces were collected from mouse cecum and weighted. PPs and ileum scrapes were homogenized and lysed in Buffer ASL (QIAGEN). Total genomic DNA from feces, PPs, and ileum scrapes were isolated using QIAamp DNA Stool Mini Kit (QIAGEN) according to the manufacturer’s instructions. The abundance of intestinal bacteria was measured by quantitative PCR on ABI prism 7500 (Applied Biosystems) using SYBR Green Master Mix (Thermo Scientific) with the same protocol described above. Specific primers to bacterial groups are 16S universal (UniF340: 5′-ACTCCTACGGGAGGCAGCAGT-3′ and UniR514: 5′-ATTACCGCGGCTGCTGGC-3′), Clostridiales (UniF338: 5′-ACTCCTACGGGAGGCAGC-3′ and C.cocR491: 5′-GCTTCTTAGTCAGGTACCGTCAT-3′), segmented filamentous bacteria (SFB) (SFB736F: 5′-GACGCTAGGCATGAGAGCAT-3′ and SFB844R: 5′-GACGGCACGGATTGTTATTCA-3′), Lactobacillaceae (LabF362: 5′-AGCAGTAGGGAATCTTCCA-3′ and LabR677: 5′-CACCGCTACACATGGAG-3′), Bacteroides (BactF285: 5′-GGTCTGAGAGGAAGGTCCC-3′ and UniR338: 5′-GCTGCCTCCCGTAGGAGT-3′), Enterobacteriaceae (Uni515F: 5′-GTGCCAGCAGCCCGCGGTAA-3′ and Ent826R: 5′-GCCTCAAGGGCACAACCTCCAAG-3′) (43), and Alcaligenes (AL1F: 5′-GGCGGACGGGTGAGTAATA-3′ and AL1R: 5′-AGTGAGAGGTCTTGCGATCC-3′) (44). The quantitative results were normalized to universal 16S rDNA and analyzed using Δ*C*_t_ method.

### Tissue Section and Immunofluorescence Assay (IFA)

For performing IFA on paraffin-embedded sections, PPs were excised, fixed in 4% paraformaldehyde at 4°C for 4 h followed by embedding in paraffin, and sectioned parallelly to the long axis of villi at 5 µm. Sections were deparaffined, rehydrated, blocked with PBS containing 1% BSA, 5% normal donkey serum, and 0.2% triton X-100, and then stained with the following antibodies: goat anti-CCL20 (R&D systems), rat anti-B220 (clone RA3-6B2, eBioscience), goat anti-IgA-biotin (SouthernBiotech), goat anti-IL-17 (polyclonal, Santa Cruz Biotechnology), and rat anti-mouse CD4-biotin (clone 4SM95, eBioscience). Sections were then washed and stained with following secondary antibodies: donkey anti-goat IgG-Alexa Fluor 594, goat anti-rat IgG-Alexa Fluor 488, and Streptavidin-Alexa Fluor 647 (Invitrogen). For performing IFA on cryosections, PPs were excised and small intestine fragments (approximately 1 cm in length) were longitudinally cut and flattened on a filter paper with mucosal side facing downwards. Tissues were then fixed in 4% paraformaldehyde at 4°C for 4 h followed by dehydration with 30% sucrose at 4°C for overnight. Tissues were embedded in tissue-tek O.C.T. compound (Sakura Finetek) and sectioned at 10 µm. PPs were sectioned parallelly to the long axis of villi and ILFs were sectioned perpendicularly to the long axis of villi. The ilea of WT and CCR6^−/−^ mice were sectioned entirely to produce consecutive sections, and sections with the largest surface areas of ILFs were subjected to IFA. Sections were fixed with ice-cold acetone, blocked with TBS containing 1% BSA, 5% normal goat serum, 5% normal donkey serum and 0.2% triton X-100, and stained with the following antibodies: rat anti-activation-induced cytidine deaminase (AID) (clone mAID-2, eBioscience), rat anti-IgD-FITC (clone 11-26c, eBioscience), rat anti-CD90.2-FITC (clone 30-H12, BioLegend), Armenian hamster anti-CD3e-biotin (clone 145-2C11, BD), rat anti-B220-PE (clone RA3-6B2, eBioscience), rat anti-RORγt (clone AFKJS-9, eBioscience), and Armenian hamster anti-CD11c-Alexa Fluor 488 (clone N418, eBioscience). Fluorescence signal of AID was amplified using TSA detection kit (PerkinElmer) according to the manufacturer’s instruction. Endogenous and excessive peroxidase activity after TSA amplification was quenched by incubating sections with 1% H_2_O_2_ solution for 10 min. Sections were then stained with the following secondary antibodies: HRP-conjugated goat anti-rat IgG (Santa Cruz Biotechnology), Streptavidin-Alexa Fluor 555, and goat anti-rat-Alexa Fluor 555 (Invitrogen). All sections were counterstained with DAPI (BioLegend), mounted in fluorescent mounting medium (Dako Cytomation), and analyzed by Zeiss LSM 700 stage confocal microscope (Carl Zeiss). The sizes of ILFs were selected manually and measured using Zen 2.1 (Carl Zeiss).

### Statistics

Statistical analysis was performed using GraphPad Prism (GraphPad Software). The Mann–Whitney *U* test was applied to evaluate the differences between two groups. Spearman correlation test was used to analyze the correlation of RORγt^+^ and B220^+^ cells with the area of a given ILF. A *P* value <0.05 was considered statistically significant.

## Results

### CCR6^−/−^ Mice Show Reduced Serum IgA and Intestinal Tissue IgA during Homeostasis

**Figure 1:**
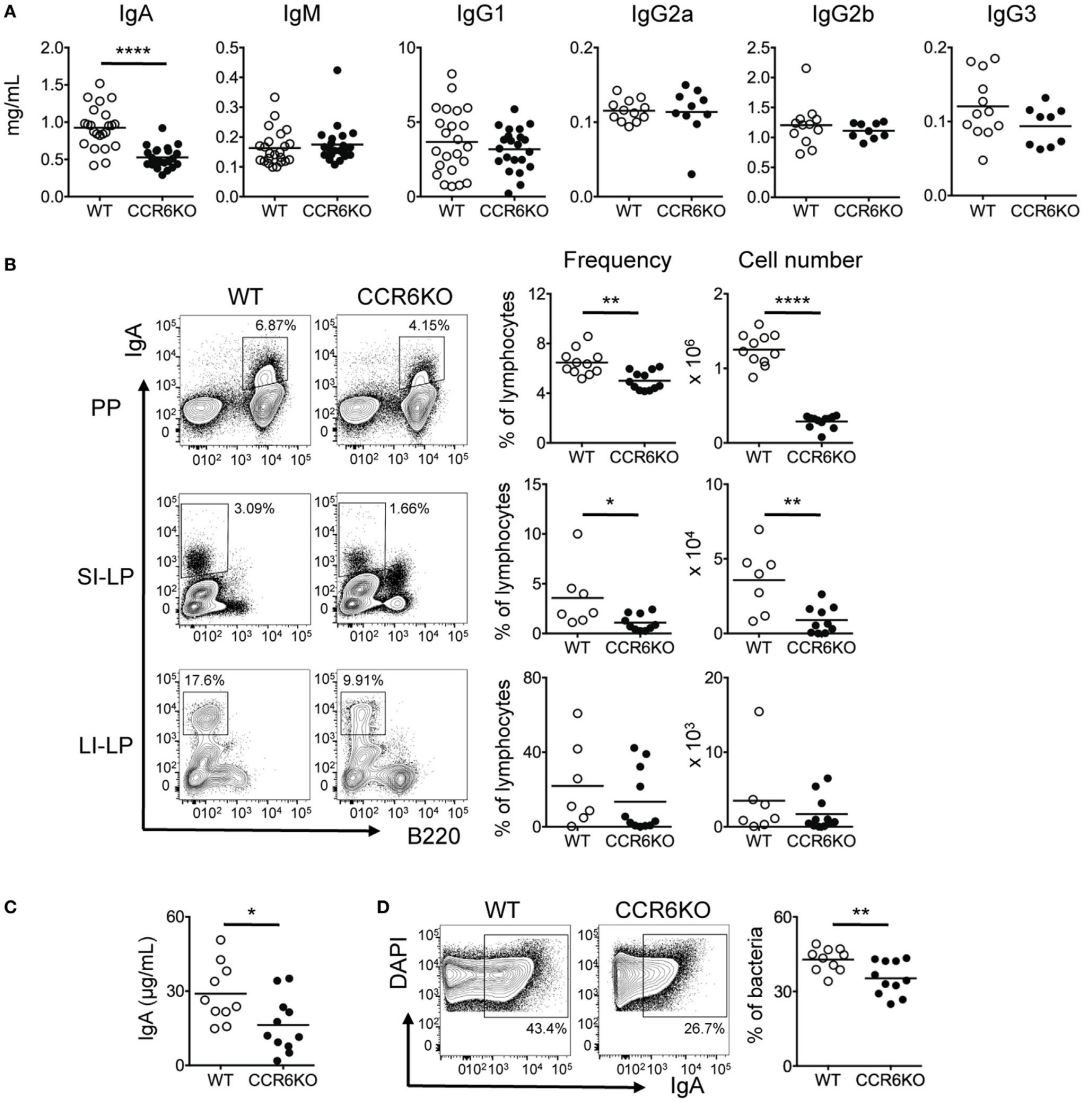
CCR6^−/−^ mice show reduced IgA production. **(A)** Sera from WT and CCR6^−/−^ littermates were analyzed for immunoglobulin subtypes using ELISA. **(B)** Lymphocytes isolated from Peyer’s patches (PPs), SI-LP, and LI-LP were subjected to FACS analysis of surface IgA and B220. The frequency and absolute number of IgA-bearing cells are shown. **(C,D)** Mouse feces were collected from the cecum, weighed, and homogenized in PBS-containing protease inhibitors. The homogenates were subjected to consecutive centrifugations to separate bacteria from bacteria-free supernatants. Bacteria-free supernatants were subjected to the analysis of IgA with ELISA **(C)**. Cecal bacteria were subjected to FACS analysis of IgA-coated bacteria **(D)**. Each symbol represents one mouse. Data are a compilation of three to five **(A)** or three independent experiments **(B–D)** (*p < 0.05; **p < 0.01; ****p < 0.0001).

To understand how CCR6 affects humoral immunity, we first examined antibody levels in serum of both WT and CCR6^−/−^ mice during homeostasis. CCR6^−/−^ mice showed significantly reduced serum IgA but comparable levels of IgM and IgG subclasses (Figure 1A). Because IgA production mainly occurs in intestinal tissues, we next examined IgA-bearing cells in various intestinal tissues. Both the frequency and number of IgA-bearing cells in PPs and small intestinal lamina propria (SI-LP) were significantly reduced in CCR6^−/−^ mice as compared to WT mice (Figure 1B). The large intestinal lamina propria (LI-LP) of CCR6^−/−^ mice also showed a trend toward reduced generation of IgA-bearing cells (Figure 1B). In addition, the levels of fecal bacteria-free IgA (Figure 1C) and the frequency of fecal bacteria-coated IgA were significantly lower in CCR6^−/−^ mice than WT mice (Figure 1D). Altogether, these results indicate that CCR6 deficiency affects IgA production during homeostasis.

### CCR6 Expressed on B Cells Plays an Intrinsic Role in TD-IgA Generation

**Figure 2:**
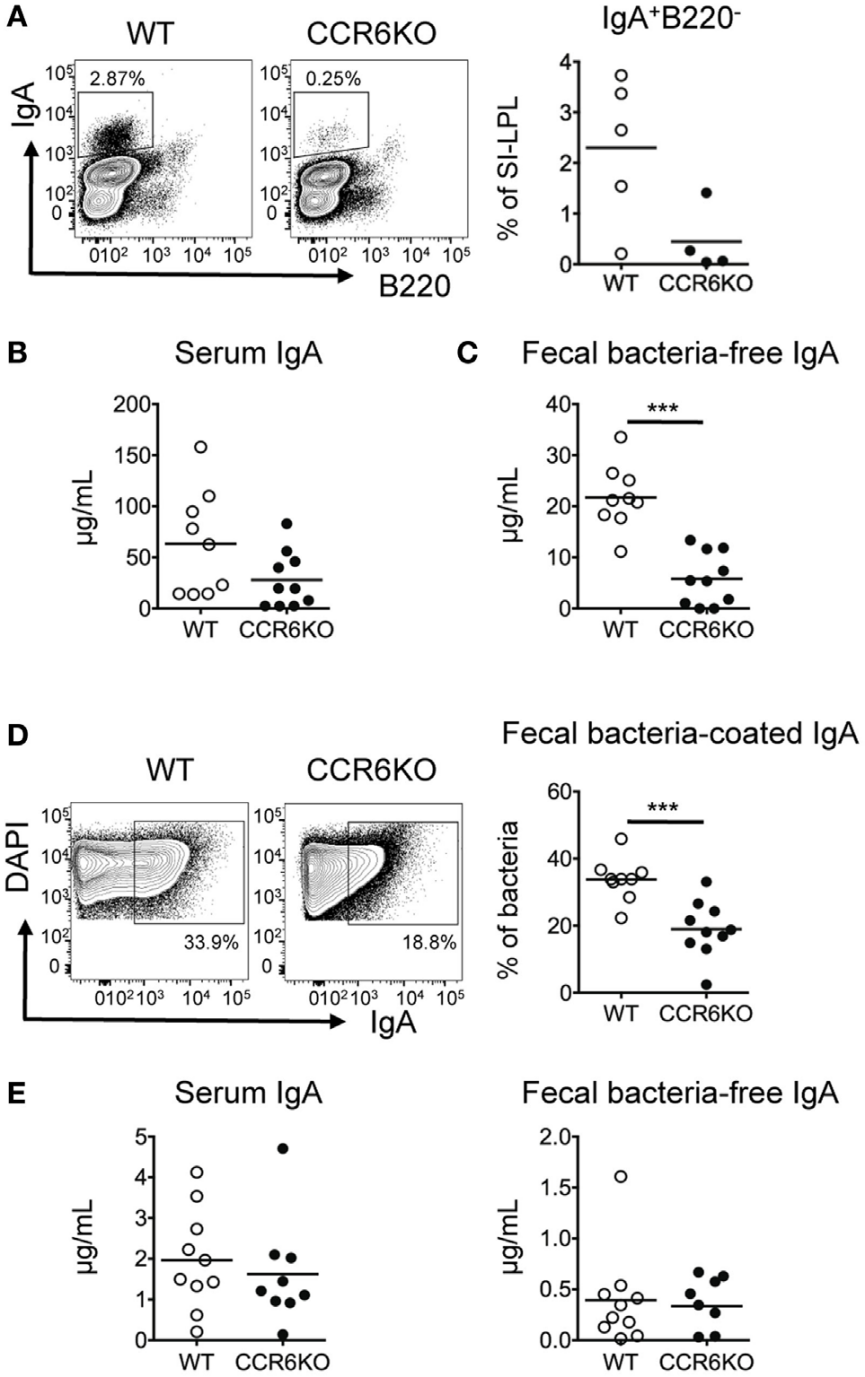
CCR6 deficiency intrinsically affects the generation of T cell-dependent IgA. Peyer’s patch (PP) naive B cells (2 × 10^6^) isolated from WT and CCR6^−/−^ mice were transferred to JH^−/−^ recipient mice *via* retro-orbital injection. **(A)** Recipient mice were sacrificed 2 weeks post-transfer, and SI-LP lymphocytes were subjected to FACS analysis of surface IgA and B220. **(B,C)** Serum **(B)** and fecal **(C)** IgA levels in the recipient mice were measured by ELISA. **(D)** Fecal bacteria were subjected to FACS analysis of IgA-coated bacteria. **(E)** PP naive B cells (2 × 10^6^) isolated from WT and CCR6^−/−^ mice were transferred to Rag1^−/−^ recipient mice. Serum IgA (left panel) and fecal IgA (right panel) were analyzed 2 weeks post-transfer. Each symbol represents one mouse. Data are a compilation of two **(A)** or three **(B–E)** independent experiments (***p < 0.001).

IgA induction mainly occurs in PPs (7). Previous studies have reported conflicting results with regard to the size of PPs in CCR6^−/−^ mice. Cook et al. reported that CCR6 deficiency did not affect the size of PPs (32), while we (Figure S1 in Supplementary Material) and others (45) find that the size of PPs in CCR6^−/−^ mice is smaller than WT. Therefore, we asked whether the diminished IgA level in CCR6^−/−^ mice (Figure 1) was due to the reduced size of PPs, containing fewer IgA-bearing B cells, or whether the IgA reduction was due to the intrinsic role of CCR6-expressing B cells in IgA production. To examine whether CCR6 expressed on B cells had an intrinsic effect on IgA production, we transferred equal numbers of naive B cells isolated from the PPs of WT or CCR6^−/−^ mice into JH^−/−^ mice or Rag1^−/−^ mice. JH^−/−^ mice receiving PP naive B cells from CCR6^−/−^ mice showed fewer IgA-bearing cells in the SI-LP (Figure 2A), reduced serum IgA (Figure 2B), and reduced fecal IgA (Figures 2C,D) compared to JH^−/−^ mice receiving PP naive B cells from WT mice. However, Rag1^−/−^ mice receiving naive B cells from the PPs of WT mice or CCR6^−/−^ mice showed similar levels of serum IgA and fecal IgA (Figure 2E). These results indicate that CCR6 expressed on B cells has an intrinsic effect on the production of TD-IgA but not T-cell-independent IgA (TI-IgA). Taken together, the results shown in Figure S1 in Supplementary Material and Figure 2 suggest that CCR6 deficiency affects IgA production by two factors. First, the IgA quantity is diminished by the development of small-sized PPs containing fewer B cells, and second, CCR6 plays an essential intrinsic role in TD-IgA production by B cells.

### CCR6^−/−^ Mice Show Enlarged GC and Impaired Production of IgA-Bearing Cells in PPs

**Figure 3:**
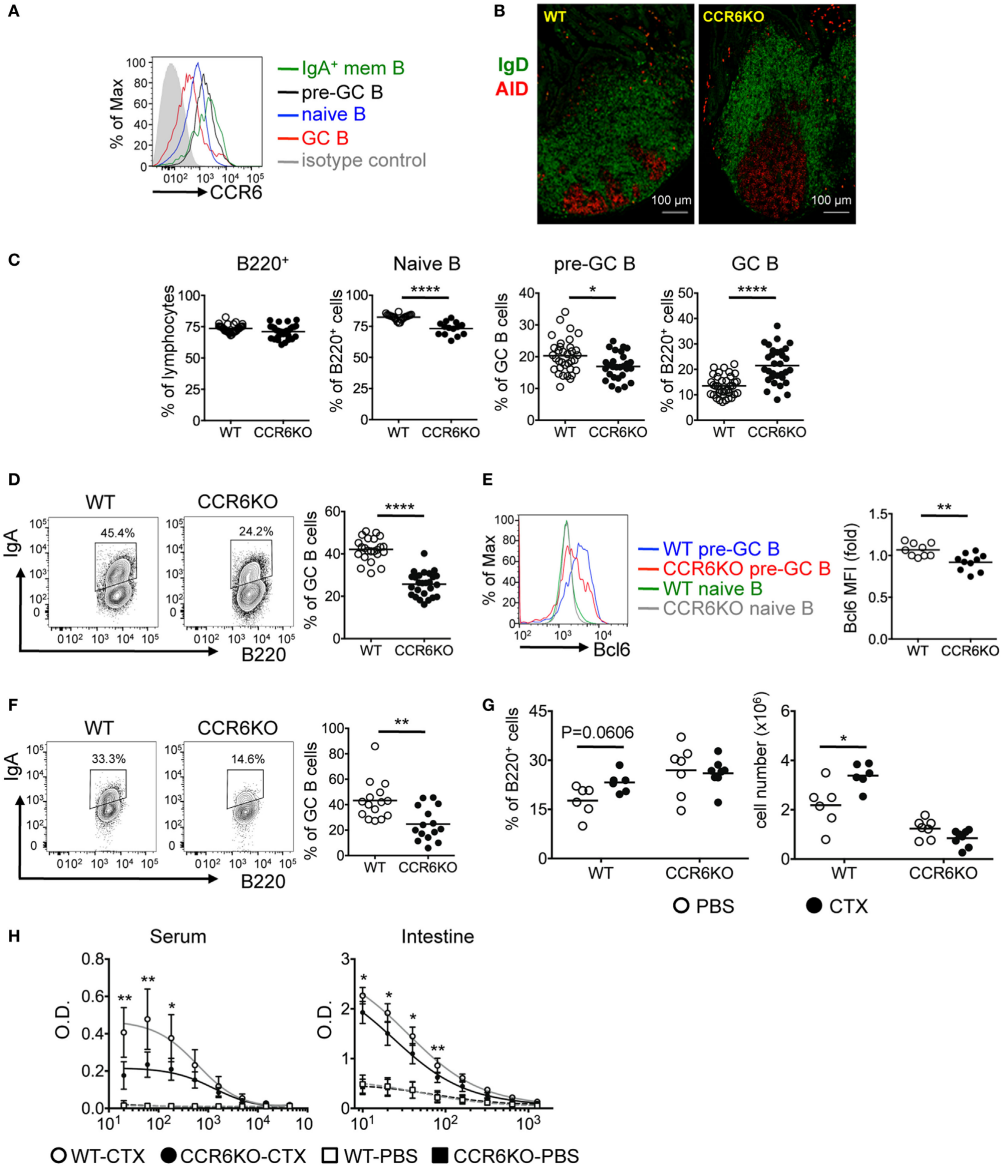
CCR6^−/−^ mice show enlarged GC and fewer IgA-bearing germinal centers (GC) B cells in Peyer’s patches (PPs). **(A)** CCR6 expression on B-cell subsets in PPs was determined by FACS analysis. B cell subsets analyzed are naive B cells (B220^+^CD38^+^CD95^−^IgD^+^), pre-GC B cells (B220^+^CD95^+^PNA^hi^IgD^+^), GC B cells (B220^+^CD95^+^PNA^hi^IgD^−^), and IgA^+^ memory B cells (B220^+^CD38^+^CD95^−^IgD^−^IgA^+^). **(B)** Cryosections of PPs from WT and CCR6^−/−^ mice. Sections were stained with AID (red) and IgD (green). PPs from two WT mice and two CCR6^−/−^ were processed and examined. Representative PPs from the distal part of small intestine are shown. **(C)** FACS analysis of B-cell subsets in PPs of WT and CCR6^−/−^ mice. The frequencies of total B cells (B220^+^), naive B cells (B220^+^CD95^−^PNA^int^IgD^+^IgM^+^), pre-GC B cells (B220^+^CD95**^+^**PNA^hi^IgD^+^), and GC B cells (B220^+^CD95**^+^**PNA^hi^) are shown. **(D)** PP lymphocytes from WT and CCR6^−/−^ mice were subjected to FACS analysis of IgA-bearing GC B cells (B220^+^CD95^+^CD38^−^IgA^+^). Representative contour plots (left panel) and the frequency (right panel) of IgA-bearing GC B cells are shown. **(E)** PP lymphocytes from WT and CCR6^−/−^ mice were subjected to FACS analysis of pre-GC B cells (B220^+^CD95^+^PNA^hi^IgD^+^) along with intracellular staining of Bcl6. A representative histogram is shown (left panel), and the mean fluorescence intensity (MFI) of Bcl6 is shown (right panel). **(F)** Irradiated Rag1^−/−^ mice were reconstituted with mixed bone marrow from WT and CCR6^−/−^ mice (1:1). Three months later, PP lymphocytes were isolated and analyzed for IgA-bearing GC B cells. Representative contour plots (left panel) and the frequency (right panel) of IgA-bearing GC B cells are shown. **(G,H)** WT and CCR6^−/−^ mice were orally immunized with 10 µg of cholera toxin (CTX) three times with 7 days intervals. Mice were sacrificed on day 7 after final immunization. PP lymphocytes were subjected to FACS analysis of GC B cells (B220^+^CD95**^+^**PNA^hi^). The frequency (left panel) and number (right panel) of GC B cells are shown **(G)**. Mouse sera were adjusted to an equal IgA concentration (100 mg/ml), and intestinal tissue extracts were adjusted to an equal protein concentration (5 mg/ml). Sera and intestinal samples were serially diluted, followed by measurement of anti-CTX IgA with ELISA **(H)**. Each symbol represents one mouse. Data are a compilation of six **(C)**, four **(D,E)**, three **(F)**, or two **(G,H)** independent experiments (*p < 0.05; **p < 0.01; ****p < 0.0001).

We next examined the expression of CCR6 on PP B-cell subpopulations. CCR6 is expressed homogeneously on naive B cells (B220^+^CD38^+^CD95^−^IgD^+^), upregulated on pre-GC B cells (B220^+^CD95^+^PNA^hi^IgD^+^), downregulated on GC B cells (B220^+^CD95^+^PNA^hi^IgD^−^), and reexpressed at the highest level on IgA-bearing memory B cells (B220^+^CD38^+^CD95^−^IgD^−^IgA^+^) (Figure 3A). Given that PPs are the major induction sites of TD-IgA (46) and that CCR6 on B cells plays an intrinsic role in TD-IgA production (Figure 2), and that CCR6^−/−^ mice showed reduced IgA (Figure 1), we hypothesized that CCR6^−/−^ mice might have abnormalities in the structure and function of PPs, leading to impaired IgA production. Examining the GC structure by IFA, we found that CCR6^−/−^ mice showed enlarged GCs as compared to WT mice (Figure 3B). This observation prompted us to analyze the subpopulations of B cells in PPs of WT and CCR6^−/−^ mice. The frequency of B cells (B220^+^) was similar between WT and CCR6^−/−^ mice. Interestingly, the frequencies of naive B cells (B220^+^CD95^−^PNA^int^IgD^+^IgM^+^) and pre-GC B cells (B220^+^CD95^+^PNA^hi^IgD^+^) were significantly decreased in CCR6^−/−^ mice (Figure 3C), whereas the frequency of GC B cells (B220^+^CD95^+^PNA^hi^) was significantly increased in CCR6^−/−^ mice as compared to WT mice (Figure 3C). The increased frequency of GC B cells may explain the enlarged GC structure in PPs of CCR6^−/−^ mice.

Interestingly, we found that while CCR6^−/−^ mice showed increased GC B cells (Figures 3B,C), the GC B cells were not frequently IgA-bearing (B220^+^CD95^+^CD38^−^IgA^+^) (Figure 3D). We hypothesized that CCR6^−/−^ mice might have a defect in generating IgA-bearing GC B cells due to the impaired IgA class switching in GC reactions. It has been reported that sustained T–B cell interactions require Bcl6 expression in pre-GC B cells to undergo GC reactions and that Bcl6 positively regulates activation-induced cytidine deaminase (AID), which is required for somatic hypermutation and class-switch recombination in GC reactions (47, 48). Thus, we examined Bcl6 expression in the pre-GC B cells of WT and CCR6^−/−^ mice and found significantly lower levels of Bcl6 expression in the pre-GC B cells of CCR6^−/−^ mice as compared to WT mice (Figure 3E). The reduced Bcl6 expression in the pre-GC B cells in CCR6^−/−^ mice may affect the sustained T–B cell interactions and GC reactions, leading to insufficient generation of IgA in CCR6^−/−^ mice and providing an explanation for the observed reduction of IgA generation in CCR6^−/−^ mice.

To further demonstrate the intrinsic effect of CCR6 on IgA-bearing GC B cells, we generated mixed bone marrow chimeras by reconstituting Rag1^−/−^ recipients with bone marrow from WT and CCR6^−/−^ mice mixed at a 1:1 ratio. Analyzing the PPs of chimeras, we found significantly fewer IgA-bearing GC B cells derived from CCR6^−/−^ bone marrow than WT bone marrow (Figure 3F), further confirming the intrinsic role of CCR6 in generating IgA-bearing GC B cells.

To test the physiological importance of CCR6 effects on immunization-stimulated IgA generation, we orally immunized WT and CCR6^−/−^ mice with cholera toxin (CTX). Interestingly, both the frequency and the absolute number of GC B cells were increased in WT mice upon oral challenge with CTX; however, GC B cells in CCR6^−/−^ mice failed to expand after challenge, even though the frequency of these cells was higher in CCR6^−/−^ mice than in WT mice during homeostasis (Figure 3G). Accordingly, the generation of CTX-specific IgA was significantly lower in both serum and the intestines of CCR6^−/−^ mice (Figure 3H).

Together, these results demonstrate that CCR6 deficiency affects GC structure and functionality in PPs, leading to impaired generation of IgA-bearing GC B cells and reduced production of antigen-specific IgA.

### The Generation of IgA-Bearing Memory B Cells in PPs Is Significantly Decreased in CCR6^−/−^ Mice

**Figure 4:**
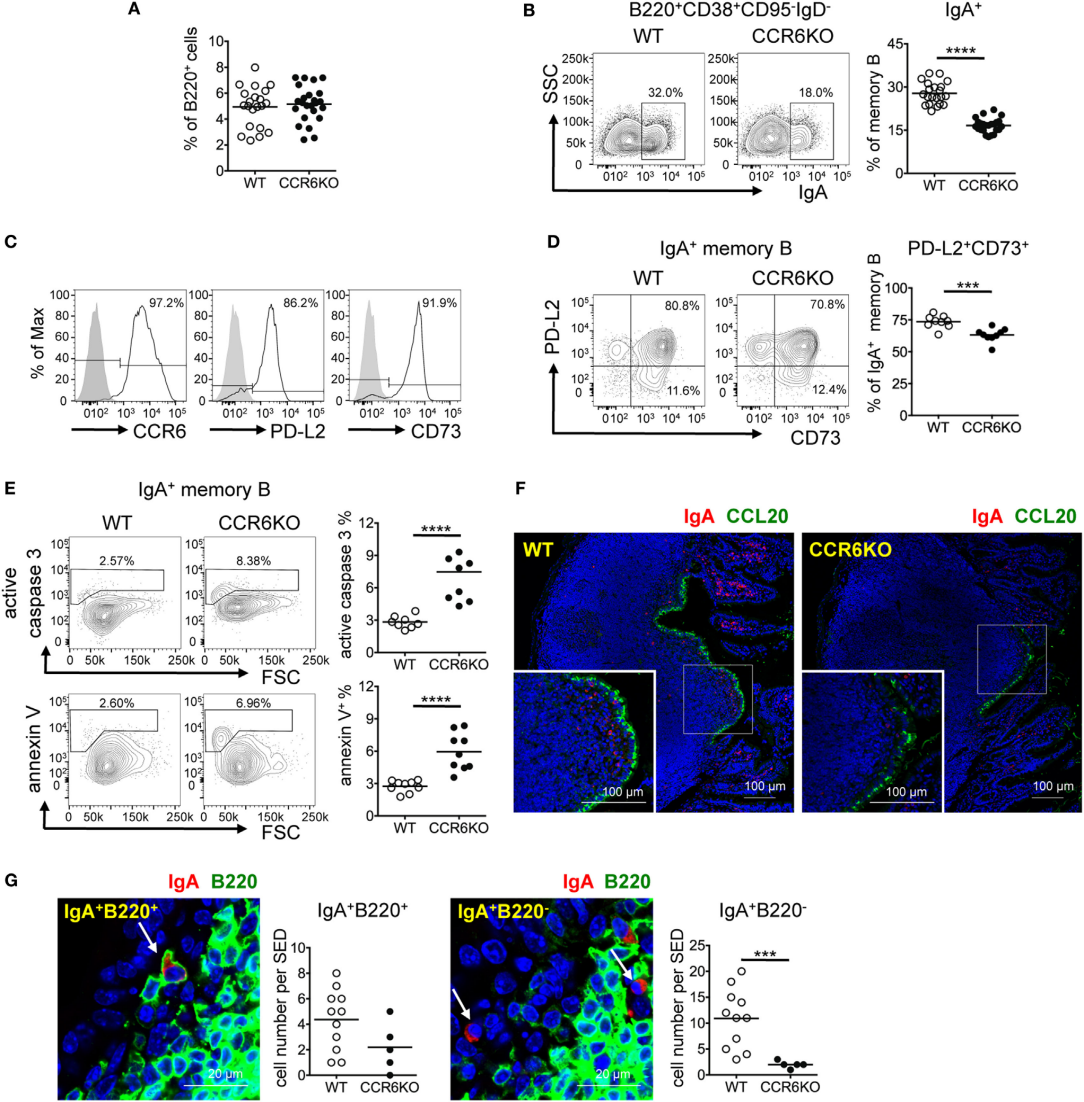
CCR6^−/−^ mice show decreased IgA-bearing memory B cells in Peyer’s patches (PPs). **(A–E)** PP lymphocytes from WT and CCR6^−/−^ mice were subjected to FACS analysis of memory B cells. The frequencies of total memory B cells (B220^+^CD38^+^CD95^−^IgD^−^) **(A)** and IgA-bearing memory B cells **(B)** are shown. Representative histograms indicating the expression of CCR6, PD-L2, and CD73 on IgA-bearing memory B cells are shown **(C)**. Representative contour plots (left panel) and the frequency of PD-L2 and CD73 double-positive, IgA-bearing memory B cells in WT and CCR6^−/−^ mice (right panel) are shown **(D)**. Representative contour plots and the frequency of IgA-bearing memory B cells positive for active caspase 3 and annexin V in WT and CCR6^−/−^ mice are shown **(E)**. **(F,G)** Sections of paraffin-embedded PPs from WT and CCR6^−/−^ mice were subjected to immunofluorescence assay (IFA) for the detection of IgA (red) and CCL20 (green) **(F)** or IgA (red) and B220 (green) **(G)**. PPs from four mice (WT *n* = 2, CCR6^−/−^ *n* = 2) were processed and examined. Representative IFAs from cryosection of the distal part of small intestine are shown **(F)**. IgA^+^B220^+^ cells (left panel) and IgA^+^B220^−^ cells (right panel) in the subepithelial dome (SED) of WT PPs are shown. The quantification of the IgA-bearing cells was performed by counting cells within the SED, which is depicted as a more diffuse area immediately underneath follicle-associated epithelium. The results obtained from one pair of WT and CCR6^−/−^ mice are shown **(G)**. Each symbol represents one mouse **(A–E)**. Each symbol represents one SED **(G)**. Data are a compilation of six **(A,B)**, two **(D)**, or three **(E)** independent experiments (***p < 0.001; ****p < 0.0001).

Knowing that CCR6^−/−^ mice showed fewer IgA-bearing GC B cells (Figures 3D,F), we expected that CCR6^−/−^ mice would produce fewer IgA-bearing memory B cells. Interestingly, WT and CCR6^−/−^ mice showed comparable frequencies of total memory B cells (B220^+^CD38^+^CD95^−^IgD^−^) in PPs (Figure 4A). However, CCR6^−/−^ mice showed significantly fewer IgA-bearing memory B cells (B220^+^CD38^+^CD95^−^IgD^−^IgA^+^) (Figure 4B). Given that both CD73 and PD-L2 expression are associated with high levels of somatic mutations in T-cell-dependent memory B cells (49), we examined CD73 and PD-L2 expression on IgA-bearing memory B cells. Almost all IgA-bearing memory B cells expressed CCR6, and the majority of IgA-bearing memory B cells were positive for PD-L2 and CD73 (Figure 4C). However, the frequency of PD-L2^+^CD73^+^ double-positive IgA^+^ memory B cells in PPs of CCR6^−/−^ mice was significantly reduced (Figure 4D), with this effect being mainly attributable to the reduced expression of CD73. Because elevated expression of CD73 has been shown to be beneficial to cell survival (50), we further examined whether the reduced CD73 expression on IgA-bearing memory B cells in CCR6^−/−^ mice might account, at least in part, for their susceptibility to apoptosis. The frequencies of caspase 3-activated cells and annexin V^+^ cells were significantly increased in IgA-bearing memory B cells in CCR6^−/−^ mice (Figure 4E), but not in IgA-bearing GC B cells or naive B cells (data not shown), suggesting that IgA memory B cells without CCR6 expression are indeed prone to apoptosis.

Interestingly, examining the anatomical localization of IgA-bearing cells in PPs using IFA, we found that there were IgA-bearing cells within the SED and that fewer IgA-bearing cells were localized within the SED in CCR6^−/−^ mice as compared to WT mice (Figure 4F). Some IgA-bearing cells within the SED were B220 positive, likely indicating that they are IgA memory B cells (IgA^+^B220^+^) (Figure 4G, left panel). We also noticed some IgA^+^ cells without B220 expression (IgA^+^B220^−^) within the SED of PPs, and the number of these cells was also reduced in CCR6^−/−^ mice (Figure 4G, right panel). Because memory B cells have the highest levels of CCR6 among B cell subsets (Figure 3A), we expected that CCL20 expressed in FAE may be important for IgA memory B cells to migrate toward and establish residence in the SED. Interestingly, despite CCR6 deficiency on IgA-bearing B cells, some IgA^+^B220^+^ and IgA^+^B220^−^ cells were still found within the SED in CCR6^−/−^ mice. This observation may be due to the non-directional migration of cells toward the SED. Altogether, these results demonstrate the important role of CCR6 in the generation, maintenance, and positioning of IgA-bearing memory B cells in the SED.

### The Milieu of PPs in CCR6^−/−^ Mice Is Altered and Becomes Unfavorable for B-Cell Homing

**Figure 5:**
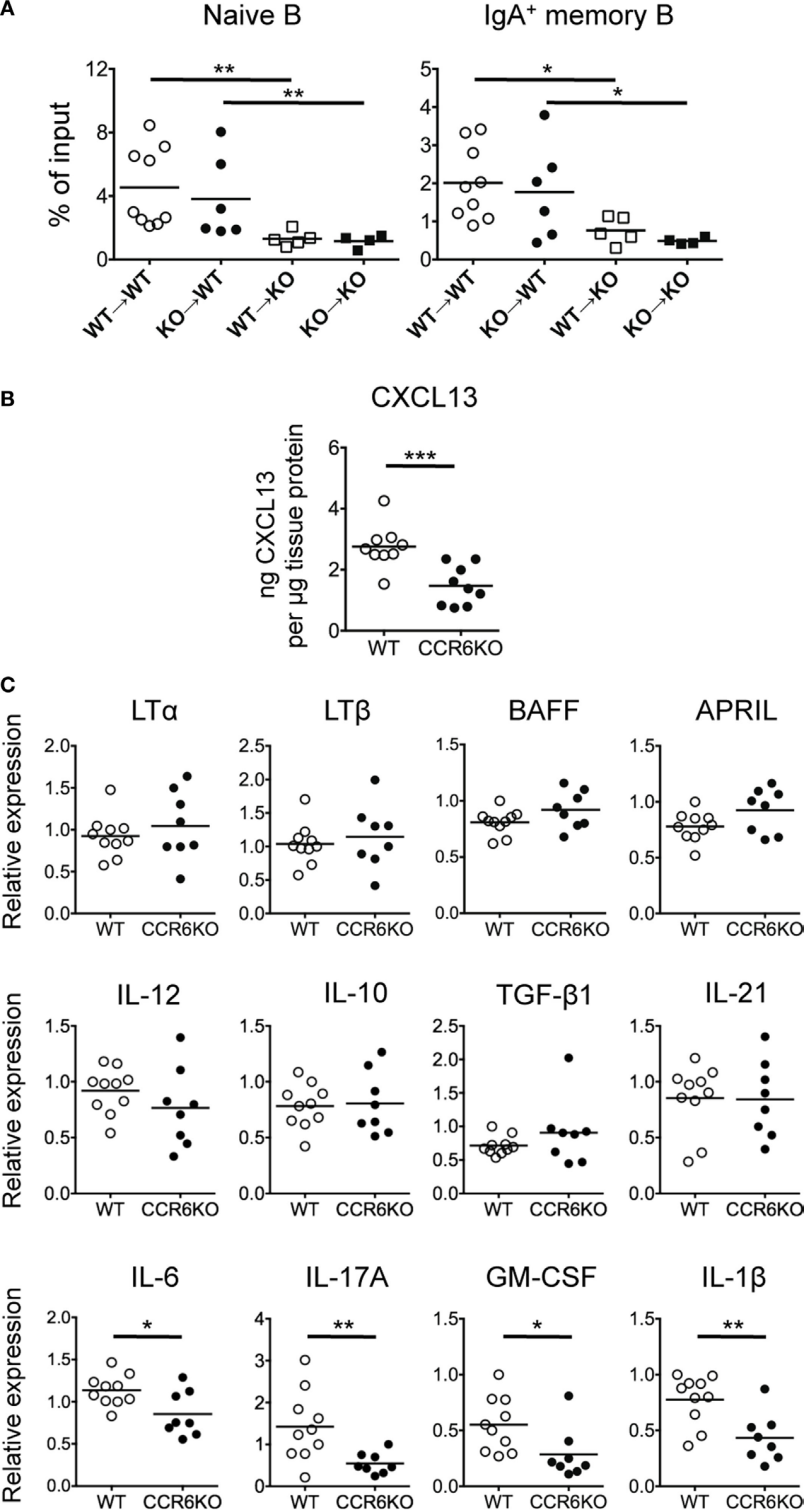
The Peyer’s patch (PP) milieu determines B cells trafficking toward PPs. **(A)** PP lymphocytes (1 × 10^7^) from WT and CCR6^−/−^ mice were adoptively transferred into recipient mice with different CD45 congenital markers. Recipient mice were sacrificed 48 h post-transfer, and PP lymphocytes were subjected to FACS analysis of donor naive B cells and donor IgA-bearing memory B cells. **(B)** Concentrations of CXCL13 in PP homogenates from WT and CCR6^−/−^ mice were determined by ELISA and normalized to the concentrations of total tissue protein. **(C)** The expression of various genes in PPs of WT and CCR6^−/−^ mice was determined by quantitative PCR. Relative gene expression was normalized to the level of GAPDH and compared to the expression in WT mice. Each symbol represents one mouse. Data are a compilation of three **(A,B)** or three to four **(C)** independent experiments (*p < 0.05; **p < 0.01; ***p < 0.001).

Because both CCR6 and CCL20 are highly expressed in PPs (32) and CCR6^−/−^ mice show small-sized PPs (Figure S1 in Supplementary Material), it has been postulated that CCR6–CCL20 axis may regulate B-cell homing to PPs (34). We next examined whether CCR6 deficiency impaired B-cell homing to PPs, leading to the small-sized PPs, and whether CCR6 deficiency altered the microenvironment of PPs, leading to the aberrant GC reactions, and subsequently reduced IgA production (Figures 1–4). We performed *in vivo* migration assays by transferring PP lymphocytes into recipient mice with different congenic markers and then analyzed donor B-cell subpopulations in PPs. Following the transfer of the same batch of PP lymphocytes from either WT or CCR6^−/−^ mice into WT or CCR6^−/−^ recipient mice, CCR6^−/−^ recipient mice showed significantly decreased migration of both naive B cells and IgA-bearing memory B cells compared to WT recipient mice (naive B cells: WT → WT vs. WT → CCR6^−/−^, *P* < 0.01; CCR6^−/−^ → WT vs. CCR6^−/−^ → CCR6^−/−^, *P* < 0.01; IgA memory B cells: WT → WT vs. WT → CCR6^−/−^, *P* < 0.05; CCR6^−/−^ → WT vs. CCR6^−/−^ → CCR6^−/−^, *P* < 0.05) (Figure 5A). These results suggest that the milieu of PPs in CCR6^−/−^ mice is important for B cells to traffic toward PPs. Interestingly, no significant difference in the migration of naive B cells and IgA memory B cells was observed when WT or CCR6^−/−^ recipients received PP lymphocytes isolated from either WT or CCR6^−/−^ mice (WT → WT vs. CCR6^−/−^ → WT; WT → CCR6^−/−^ vs. CCR6^−/−^ → CCR6^−/−^) (Figure 5A). These results indicate that CCR6 expression on B cells is dispensable for B cell migration toward PPs. This result is in contrast with previous proposal that CCR6 on B cells plays a critical role in B cells homing to PPs (34). Instead, our results demonstrate that CCR6 deficiency leads to alterations in the PP milieu, which impairs B cell homing to PPs.

### CCR6^−/−^ Mice Show Significantly Reduced Expression of Th17-Related Cytokines in PPs

Knowing that the milieu of PPs is altered in CCR6^−/−^ mice (Figure 5A), we next examined whether the expression levels of B cell-associated cytokines and chemokines were altered. CXCL13, a potent B cell chemoattractant, was significantly reduced in PPs of CCR6^−/−^ mice (Figure 5B), suggesting that CXCL13 may be a critical factor driving B cell migration toward PPs, consistent with a previous report by Okada et al. (51). Cytokines related to B cell differentiation into IgA-producing cells (TGF-β, IL-10, IL-21, BAFF, and APRIL) and cytokines related to lymphoid organogenesis (LTα and LTβ) were not significantly altered in PPs of WT and CCR6^−/−^ mice (Figure 5C). Interestingly, cytokines (IL-6, IL-17, GM-CSF, and IL-1β) related to Th17 cell differentiation and secretion were reduced in PPs of CCR6^−/−^ mice (Figure 5C), suggesting that CCR6 deficiency affects the function and differentiation of Th17 cells in PPs. These results support the conclusion that CCR6 deficiency alters the milieu of PPs, which may affect PP functionality.

### CCR6^−/−^ Mice Show Reduced Frequency of Th17 Cells in PPs

**Figure 6:**
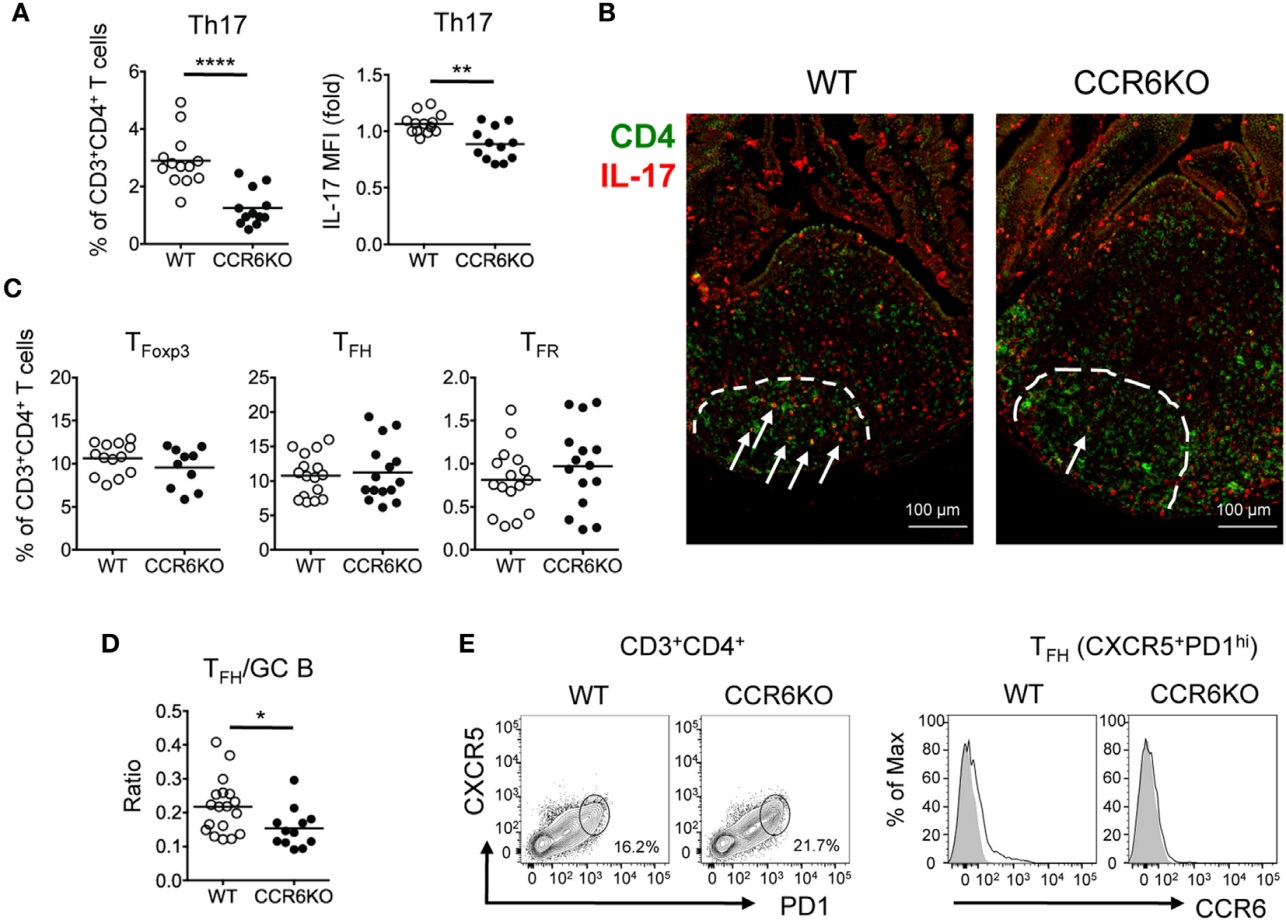
CCR6^−/−^ mice show significantly reduced Th17 cells in Peyer’s patches (PPs). **(A)** PP lymphocytes were subjected to FACS analysis of Th17 cells (CD3^+^CD4^+^IL-17^+^). The frequency of Th17 in T helper cells (left panel) and the mean fluorescence intensity of IL-17 in Th17 cells (right panel) are shown. **(B)** Representative immunofluorescence assay of CD4 (green) and IL-17 (red) from paraffin-embedded PPs of WT and CCR6^−/−^ mice are shown. Dash lines indicate GC boundary. Arrows indicate CD4^+^IL-17^+^ double-positive cells within germinal centers (GC). **(C)** FACS analysis of PP lymphocytes. The frequencies of T_Foxp3_ (CD3^+^CD4^+^Foxp3^+^), T_FH_ (CD3^+^CD4^+^CXCR5^+^PD1^+^), and T_FR_ (CD3^+^CD4^+^CXCR5^+^PD1^+^Foxp3^+^) cells are shown. **(D)** PP lymphocytes from WT and CCR6^−/−^ mice were prepared and subjected to FACS analysis for detecting T_FH_ (CD3^+^CD4^+^CXCR5^+^PD1^+^) and GC B (B220^+^CD95^+^PNA^hi^) cells. The absolute cell number of T_FH_ and GC B cells was calculated. The ratio of T_FH_/GC B was calculated by dividing the absolute cell number of T_FH_ cells by the absolute cell number of GC B cells. **(E)** Representative contour plots of T_FH_ cells (CD3^+^CD4^+^CXCR5^+^PD1^+^) in PPs (left panel) and representative histograms of CCR6 expression on T_FH_ cells (right panel) are shown. Each symbol represents one mouse. Data are a compilation of six **(A)**, four **(C)**, or three **(D)** independent experiments (**p < 0.01; ****p < 0.0001).

Since we found that IL-17 level is significantly reduced in PPs of CCR6^−/−^ mice (Figure 5C) and it is known that Th17 cells play a role in GC reactions (52, 53), we hypothesized that the reduced level of IL-17 in CCR6^−/−^ mice might result, in part, from the reduced frequency and number of Th17 cells, which impair GC reactions in PPs and affect IgA production. We analyzed the frequency of T helper cell (CD3^+^CD4^+^) subsets in PPs. CCR6^−/−^ mice exhibited significant decreases in both the frequency of Th17 cells (CD3^+^CD4^+^IL-17^+^) (Figure 6A, left panel) and also the level of IL-17 in Th17 cells (Figure 6A, right panel). Interestingly, we observed that some CD4^+^IL-17^+^ cells were located in PP GCs, but that the number and IL-17 staining intensity of these cells were reduced in CCR6^−/−^ GCs (Figure 6B), indicating an important role for PP Th17 cells in supporting GC responses. The frequencies of T_Foxp3_ cells (CD3^+^CD4^+^Foxp3^+^), T_FH_ cells (CD3^+^CD4^+^PD1^+^CXCR5^+^), and T_FR_ cells (CD3^+^CD4^+^PD1^+^CXCR5^+^Foxp3^+^) were similar in the PPs of WT and CCR6^−/−^ mice (Figure 6C). However, the ratio of T_FH_ cells to GC B cells in the PPs of CCR6^−/−^ mice was significantly decreased as compared to WT mice (Figure 6D), providing an explanation for the observed impairment of GC reactions in CCR6^−/−^ mice. Of note, CCR6, the signature chemokine receptor for Th17 cells, was detected on some T_FH_ cells (Figure 6E) in WT PPs, supporting the notion that T_FH_ cells are likely derived from Th17 cells. Altogether, these results suggested that CCR6 deficiency specifically affects the frequency and function of Th17 cells in PPs, which may impair Th17 plasticity toward T_FH_, and consequently reduces the production of TD-IgA.

### ILC3s in PPs of CCR6^−/−^ Mice Show Reduced IL-17 and Increased MHCII Expression

**Figure 7:**
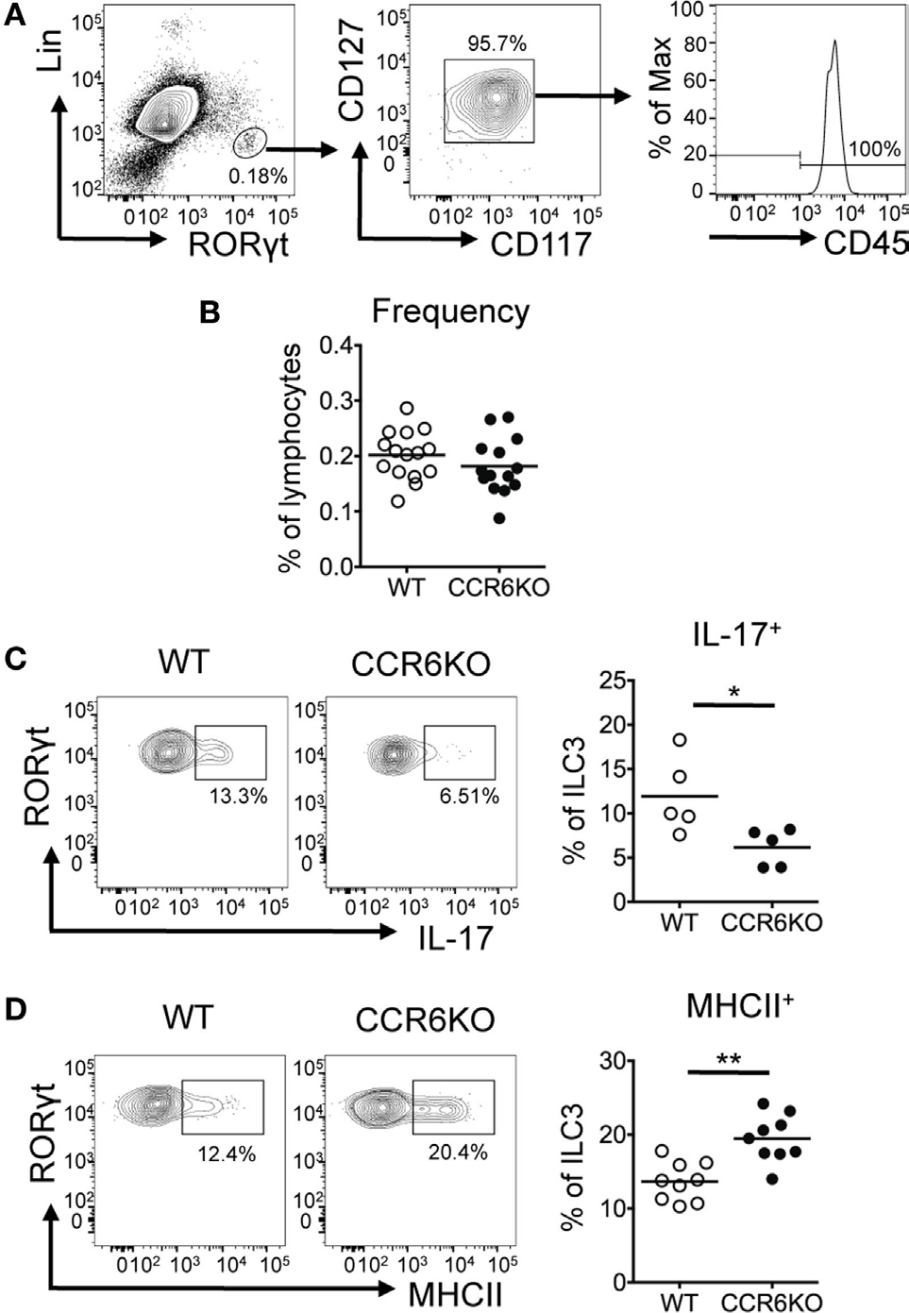
ILC3s show significantly decreased IL-17 expression but significantly increased MHCII expression in Peyer’s patches (PPs) of CCR6^−/−^ mice. PP lymphocytes were subjected to immunofluorescence staining of surface markers (Lin, CD45, CD117, CD127, and MHCII) followed by intracellular staining of RORγt. **(A)** Representative contour plots for the identification of ILC3–LTi (Lin^−^RORγt^+^CD117^+^CD127^+^) in PPs are shown. **(B)** The frequency of ILC3–LTi in PPs of WT and CCR6^−/−^ mice is shown. **(C)** PP lymphocytes were stimulated with 20 µg/ml PMA, 1 µM ionomycin, and 5 µg/ml brefeldin A for 4 h followed by surface staining of ILC3–LTi and intracellular staining of IL-17 and RORγt. The frequency of IL-17-producing ILC3–LTi in PPs of WT and CCR6^−/−^ mice is shown. **(D)** The frequency of MHCII-expressing ILC3–LTi in PPs of WT and CCR6^−/−^ mice is shown. Each symbol represents one mouse. Data are a compilation of six **(B)**, three **(C)**, or four **(D)** independent experiments (*p < 0.05; **p < 0.01).

In addition to Th17 cells, ILC3–LTi cells, a subset of ILC3s, express CCR6 and produce IL-17 (54). Since ILC3–LTi cells are known to be important in PP formation (55) and PPs in CCR6^−/−^ mice are small in size, we then examined the frequency of ILC3–LTi cells (Lin^−^CD45^+^CD127^+^CD117^+^RORγt^+^) in PPs. Although CCR6 deficiency did not affect the frequency of ILC3–LTi (Figures 7A,B), the frequency of IL-17-positive ILC3–LTi cells in PPs was significantly reduced in CCR6^−/−^ mice (Figure 7C). This result suggests that CCR6 signaling in ILC3–LTi cells may regulate IL-17 expression. The impaired production of IL-17 by ILC3–LTi cells may also contribute to the reduced IL-17 expression in PPs of CCR6^−/−^ mice as well as the reduction of Th17 cells (Figures 5C and 6A). A recent report has shown that ILC3–LTi cells, expressing high levels of MHCII, induce cell death of activated commensal bacteria-specific CD4^+^ T cells (56). We further examined whether CCR6 deficiency affected the frequency of MHCII^+^ ILC3–LTi cells in PPs. The frequency of MHCII^+^ ILC3–LTi cells in PPs was significantly higher in CCR6^−/−^ mice than that of WT mice (Figure 7D). The inverse association between the frequency of Th17 cells and the frequency of MHCII-positive ILC3–LTi in CCR6^−/−^ PPs (Figures 6A and 7D) may suggest that the decrease of Th17 cells in PPs is due, in part, to the elimination of Th17 cells by MHC-positive ILC3–LTi cells. As such, this mechanism would contribute to generating the observed reduction of Th17 cells in the PPs of CCR6^−/−^ mice, in addition to the previously described impairment of Th17 cells trafficking toward PPs (57).

### CCR6^−/−^ Mice Show Reduced IL-22 Production in the Lamina Propria, Leading to the Reduced Production of AMPs

**Figure 8:**
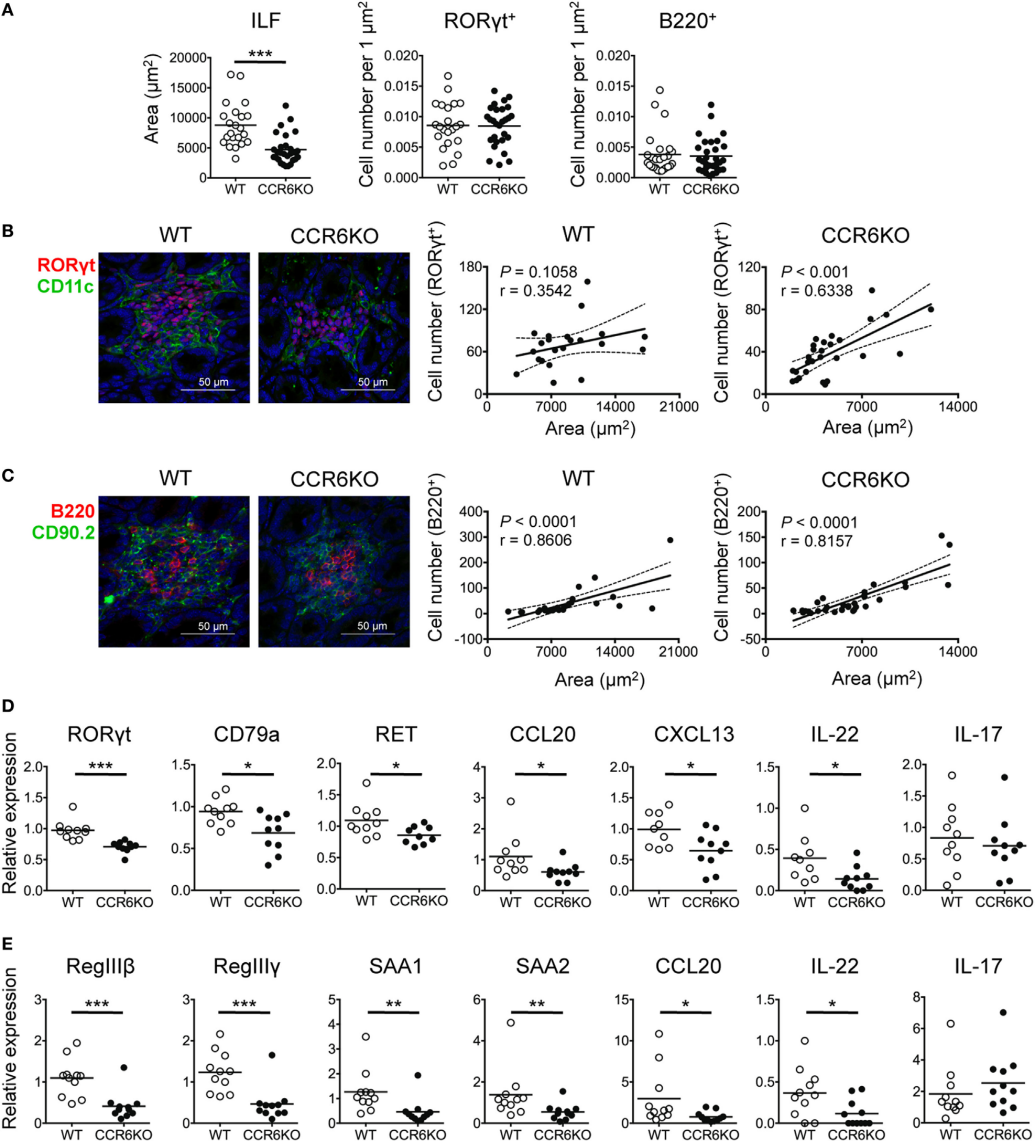
CCR6^−/−^ mice show small-sized isolated lymphoid follicles (ILFs) and reduced expression of genes involved in epithelial defense. **(A–C)** Cryosections of ileum from WT and CCR6^−/−^ mice were subjected to immunofluorescence assay (IFA) to detect ILFs. ILFs were defined as lymphoid aggregates with surface areas between 2,000 and 20,000 µm^2^. ILFs were stained for RORγt (red) and CD11c (green) [**(B)**, left panel] or B220 (red), CD90.2 (green), and CD3e (no signal, data not shown) [**(C)**, left panel]. Quantification of ILF surface areas [**(A)**, left panel] and the frequencies of RORγt^+^ cells and B220^+^ cells in ILFs are shown [**(A)**, middle and right panels]. **(B,C)** Representative IFA of ILFs in the ilea of WT and CCR6^−/−^ mice are shown [**(B,C)**, both left panel]. The scatter plots show the correlation between RORγt-expressing cells and ILF surface area in WT [**(B)**, middle panel] or CCR6^−/−^ [**(B)**, right panel] mice. The scatter plots show the correlation between B220-expressing cells and ILF surface area in WT [**(C)**, middle panel] or CCR6^−/−^ [**(C)**, right panel] mice. The number of RORγt^+^ cells or B220^+^ cells was counted in a given area of tissue sections from two mice in each group; the data shown are the results of Spearman correlation test with regression line (solid line), 95% confidence interval (dashed line), *P* value, and correlation coefficient (*r*). Each symbol represents one ILF. **(D,E)** Total RNA extracted from ileum scrapes and crypts of WT and CCR6^−/−^ mice was subjected to reverse transcription into cDNA followed by quantitative PCR analysis. Relative gene expression was normalized to the level of GAPDH and compared to expression in WT mice. The expression of various genes in crypts **(D)** and scrapes **(E)** is shown. Each symbol represents one mouse. Data are a compilation of three **(D)** or four **(E)** independent experiments (*p < 0.05; **p < 0.01; ***p < 0.001; ****p < 0.0001).

In addition to IgA, AMPs that are produced by IECs upon IL-22 stimulation also play a critical role in maintaining gut homeostasis (58). ILC3–LTi cells are important for the development of intestinal cryptopatches and ILFs and are the major source of IL-22 (14, 58). Given that ILC3–LTi cells and B cells are important components of ILFs and both express CCR6, we examined whether CCR6 deficiency had an effect on the structure and functionality of ILFs. We found that the ILFs in the ileum of WT and CCR6^−/−^ mice were approximately 2,000–20,000 µm^2^. The ILFs of CCR6^−/−^ mice were mostly small-sized follicles (<5,000 µm^2^) (Figure 8A, left panel). Interestingly, WT and CCR6^−/−^ mice showed comparable frequencies of ILC3–LTi (RORγt^+^) and B cells (B220^+^) in the given ILF areas (Figure 8A, middle and right panels). The number of ILC3–LTi cells was positively correlated with the ILF area in CCR6^−/−^ mice, but not in WT mice (Figure 8B). It is possible that the ILFs in WT mice were mostly large sized (>5,000 µm^2^), in which the number of ILC3s was already saturated. Unlike ILC3–LTi cells, the number of B cells was correlated with the ILF area in both WT and CCR6^−/−^ mice (Figure 8C), indicating that CCR6 deficiency does not affect the initial B-cell trafficking toward ILFs, but causes ILF expansion to fail at a later stage. Notably, the reduced expression of CCL20 and CXCL13 in the ileum crypts of CCR6^−/−^ mice (Figure 8D) is reminiscent of the reduced expression of CCL20 and CXCL13 in PPs of CCR6^−/−^ mice (Figures 4F and 5B). The small-sized ILF is a recapitulation of small-sized PP in CCR6^−/−^ mice, indicating that both follicular structures may suffer from a defect of B cell influx or maintenance at the later stage.

Furthermore, the expression of RORγt (a marker of ILC3s and Th17 cells) and CD79a (a marker of B cells) was significantly reduced in the crypts of CCR6^−/−^ mice (Figure 8D). Because we did not observe differences in IL-17 levels in the ileum scrapes and crypts between WT and CCR6^−/−^ mice (Figures 8D,E), we presumed that the decreased RORγt in the crypts of CCR6^−/−^ mice resulted from the reduced number of ILC3–LTi cells rather than from Th17 cells. A recent report showed that the stimulation of neuroregulatory receptor RET on ILC3–LTi cells is critical for ILC3–LTi cells to produce IL-22 (59). Notably, we found that the reduced RET expression in the crypts of CCR6^−/−^ mice was associated with the diminished IL-22 levels (Figure 8D). The reduced number of ILC3–LTi cells and the reduced level of RET in CCR6^−/−^ mice would attenuate IL-22 production and subsequently impair AMP production. The levels of antimicrobial lectins, regenerating islet-derived protein III (RegIII) and the serum amyloid A family, which was recently shown to be important for protecting hosts from microbial infection (60), were significantly reduced in the IECs of CCR6^−/−^ mice (Figure 8E). These results suggest that CCR6 deficiency precipitates the underdevelopment of ILFs with fewer B cells and ILC3–LTi cells in the intestines. The reduced numbers of ILC3–LTi cells in CCR6^−/−^ mice lead to less IL-22 production and subsequently less AMP production.

### CCR6^−/−^ Mice Have an Altered Composition of Commensal Bacteria

**Figure 9:**
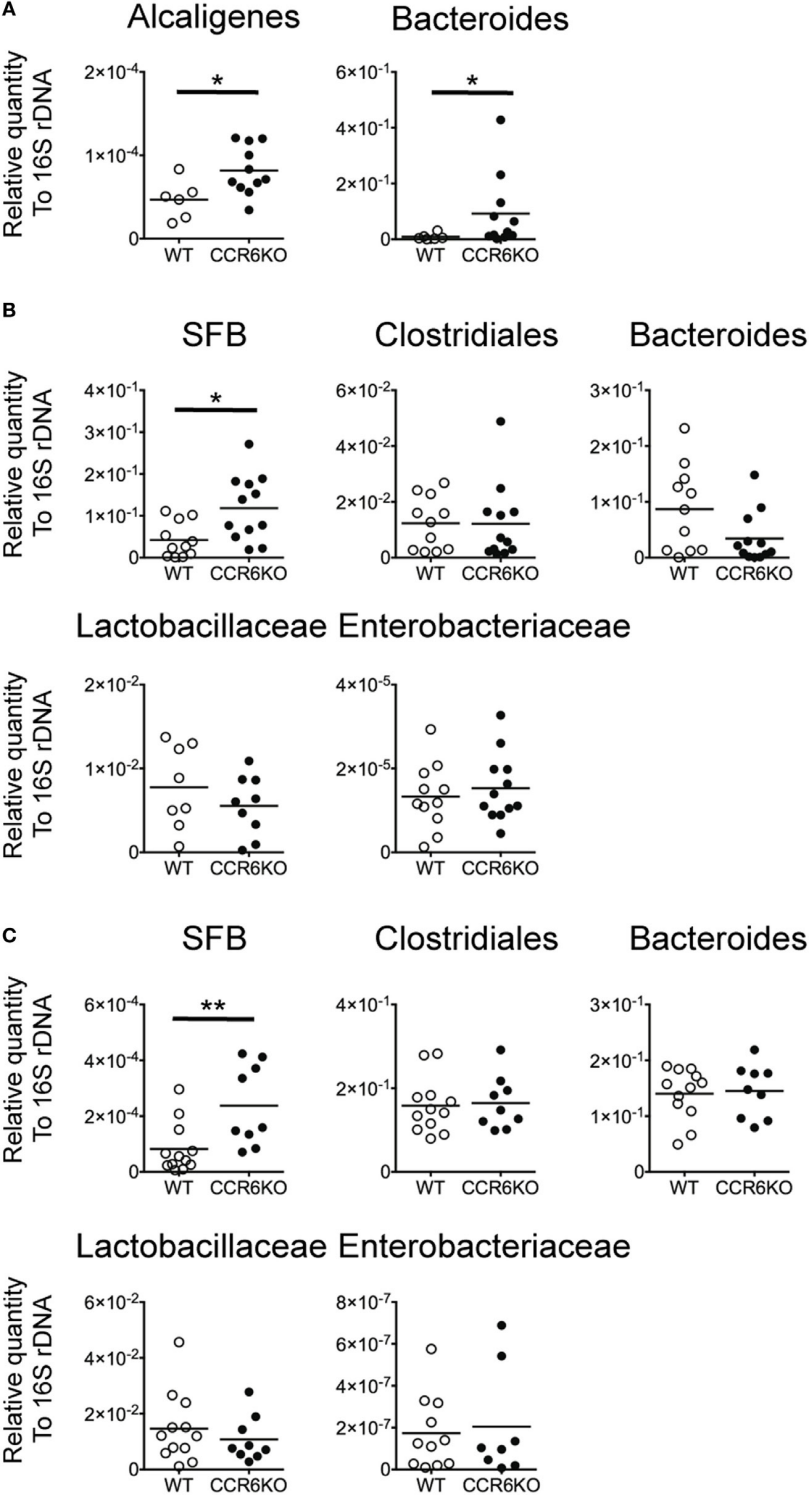
CCR6 deficiency results in the altered intestinal flora. Peyer’s patches (PPs), ileal scrapes, and cecal feces were collected from WT and CCR6^−/−^ mice. Genomic DNA was purified from the tissues and feces. The levels of indicated bacteria species were analyzed by quantitative PCR using primers specific to *Alcaligenes, Bacteroides, Clostridiales*, segmented filamentous bacteria, *Lactobacillaceae*, and *Enterobacteriaceae*. The quantitative results were normalized to universal 16S rDNA. The levels of indicated bacterial species in PPs **(A)**, ileal scrapes **(B)**, and feces **(C)** are shown. Each symbol represents one mouse. Data are a compilation of three **(A,B)** or four **(C)** independent experiments (*p < 0.05; **p < 0.01).

Both IgA and AMPs are important for maintaining intestinal homeostasis (61, 62). We hypothesized that since CCR6^−/−^ mice show lower levels of IgA (Figures 1–4) and reduced AMP production (Figure 8), they might exhibit an aberrant composition of commensal bacteria in homeostatic conditions. We examined the composition of commensal bacteria by analyzing 16S rDNA from PPs, the ileum, and feces by quantitative PCR. CCR6^−/−^ mice showed significantly increased levels of *Alcaligenes*, which are intra-tissue commensal bacteria that exclusively inhabit PPs (Figure 9A) (63). Notably, *Bacteroides* are not expected to be present in PPs, but CCR6^−/−^ mice displayed an abundance of *Bacteroides* in PPs (Figure 9A). In addition, CCR6^−/−^ mice showed significantly more SFB, but not other species, in ileal epithelial cells (Figure 9B) and feces (Figure 9C). These results demonstrate that CCR6 deficiency leads to an altered composition of commensal bacteria in intestinal tissues, which subsequently disrupts intestinal homeostasis.

## Discussion

**Figure 10:**
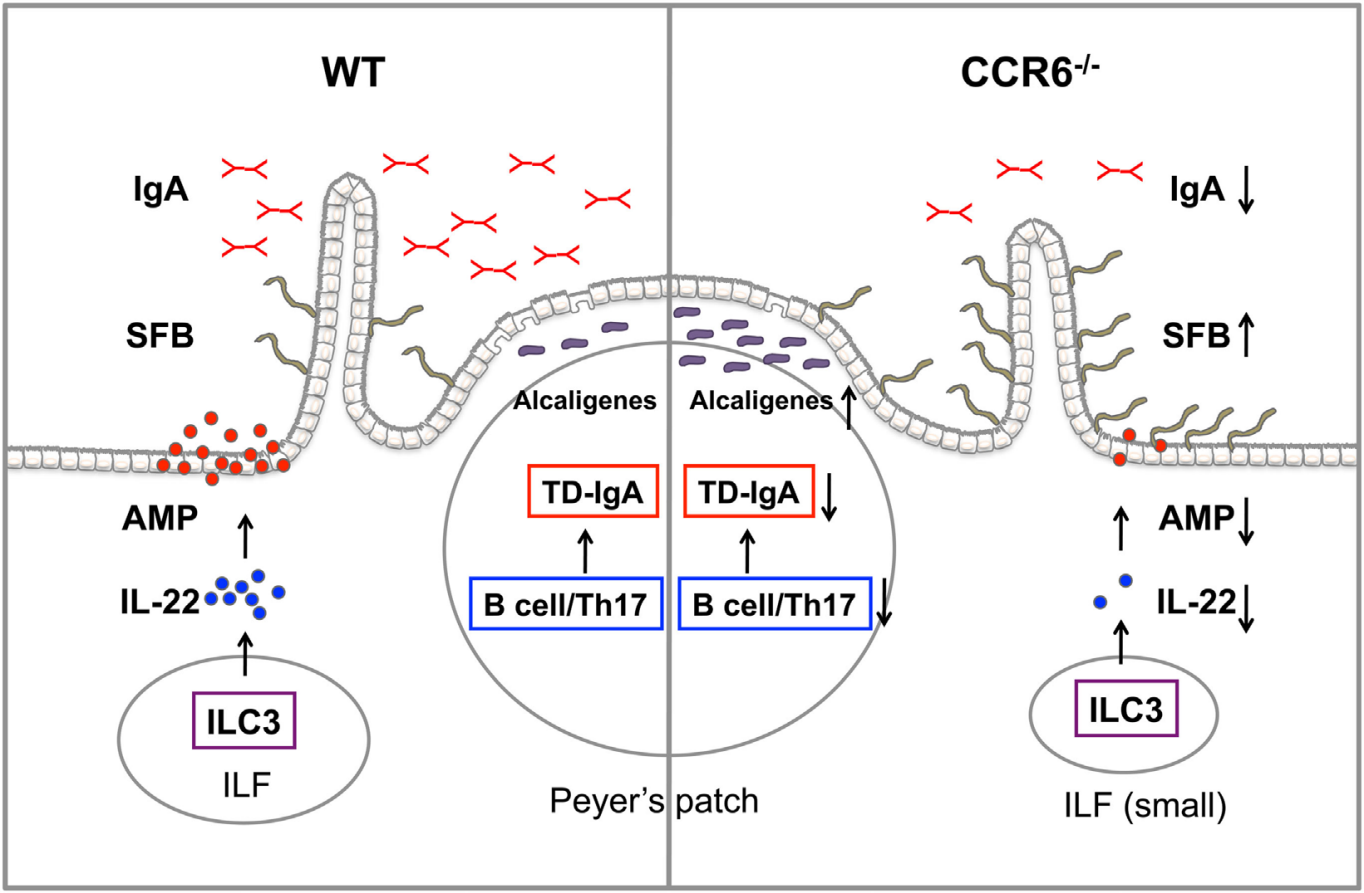
The summary of CCR6 deficiency leading to the altered intestinal flora. CCR6 deficiency affects T-cell-dependent IgA (TD-IgA) production in PPs and antimicrobial peptide (AMP) production in intestinal epithelial cells, leading to an increase of segmented filamentous bacteria and *Alcaligenes*, and subsequently to the perturbations of intestinal homeostasis.

In this study, we demonstrate that the CCR6–CCL20 axis is critical for both adaptive and innate immunity to maintain intestinal homeostasis. CCR6 plays an important role in humoral immunity, and CCR6 deficiency intrinsically affects the functionality of B cells and Th17 cells, impairs GC reactions, and subsequently leads to impaired TD-IgA production. In contrast, CCR6 is critical for innate immune responses mediated by ILC3–LTi cells. CCR6 deficiency impairs IL-22 production by ILC3–LTi cells, thus affecting the capability of IECs to produce AMPs. Consequently, the reduced production of IgA and AMPs in CCR6^−/−^ mice fails to maintain intestinal homeostasis and leads to an increased number of mucosa-associated commensal bacteria residing in intestinal tissues and subsequently disturbing intestinal homeostasis (Figure 10).

CCR6 and CCL20 are highly expressed in PPs, which is the major site for IgA induction (7). We have demonstrated that CCR6 has an intrinsic role in GC reactions (Figure 3F), and that CCR6 deficiency impairs TD-IgA production, but not TI-IgA production (Figure 2). T_FH_ cells, which provide help to B cells, are essential for the establishment of GC reactions to generate high-affinity TD antibodies (64). It has been shown that the plasticity of Th17 cells in development toward T_FH_ cell fate is restiricted to the PP environment and is responsible for the induction of TD-IgA responses (53). By IFA, we detected CD4^+^IL-17^+^ cells in the GC region of PPs (Figure 6B), presumably representing T_FH_ cells derived from Th17 cells. Furthermore, the number of CD4^+^IL-17^+^ cells and the expression of IL-17 are decreased in CCR6^−/−^ mice (Figure 6B). By FACS analysis, we have demonstrated that CCR6^−/−^ mice display significant reductions, not only in the frequency of Th17 cells but also in the capability of IL-17 production per cell base in PPs (Figure 6A). It is plausible that the reduced frequency and functionality of Th17 cells in PPs of CCR6^−/−^ mice may significantly affect the plasticity of Th17 cells toward T_FH_ cells, limiting help to B cells undergoing GC reactions and subsequently impairing the generation of high-affinity TD-IgA. Because CCR6 is a signature chemokine receptor for Th17 cells, it has been proposed that the reduced frequency of Th17 cells in PP of CCR6^−/−^ mice is due to the impaired recruitment of Th17 cells toward PPs (57). In our study, we found that the milieu of PPs in CCR6^−/−^ mice is altered, with cytokines (IL-6, IL-1β) involved in Th17 cell polarization being reduced. The decrease of the Th17 frequency in CCR6^−/−^ mice may be due, in part, to the alteration of PP milieu, resulting in reduced Th17 generation. Furthermore, MHCII-positive ILC3s can eliminate commensal bacteria-specific CD4^+^ T cells (56). We found an inverse association between the frequency of Th17 cells and the frequency of MHCII-positive ILC3s in PPs of CCR6^−/−^ mice (Figures 6A and 7D), which led us to propose that the elimination of Th17 cells by MHCII-positive ILC3–LTi cells may account, in part, for the observed reduction in Th17 frequency. It is plausible that the reduction of Th17 cells in PPs of CCR6^−/−^ mice may result from decreased Th17 cell generation and/or the increased elimination of Th17 cells by ILC3s with high MHCII expression. These mechanisms would be in addition to the impaired recruitment of Th17 cells into PPs as shown by Wang et al. (57).

Intriguingly, CCR6^−/−^ mice have a higher frequency of GC B cells, but a lower frequency of IgA-bearing GC B cells as compared to WT mice during homeostasis (Figure 3D). Furthermore, CCR6^−/−^ mice have increased GC B cells, but the GC B cells fail to expand and produce lower levels of high-affinity TD-IgA upon oral challenge with antigens (Figures 3G,H). All these results indicate that CCR6 plays a critical role in GC reactions for IgA class switching and somatic hypermutation. Given that Bcl6 expression in pre-GC B cells is critical for the sustained interaction of pre-GC B cells with cognate T helper cells (47) and that Bcl6 is important on the regulation of class switching and somatic hypermutation during GC reactions (48), our finding of reduced expression of Bcl6 in pre-GC B cells of CCR6^−/−^ mice (Figure 3E) may provide an explanation for the impaired generation of high-affinity TD-IgA in CCR6^−/−^ mice. It is plausible that the reduced expression of Bcl6 in the pre-GC B cells of CCR6^−/−^ mice may impede sufficient cognate interactions between pre-GC B cells and T helper cells and cause the pre-GC B cells of CCR6^−/−^ mice to aberrantly enter into GC, where they fail to efficiently undergo somatic hypermutation, thus increasing GC B cells and producing low-affinity IgA. How CCR6 signaling in B cells regulates Bcl6 expression in pre-GC B cells warrants further investigation.

Notably, the cytokine milieu in PPs of CCR6^−/−^ mice is different from that of WT mice (Figures 5B,C). It is known that cytokines are important for regulating antibody production. However, the expression of cytokines (TGF-β, IL-10, and IL-21) involved in IgA class switching is not affected by CCR6 deficiency. We noted that IL-6 expression in PPs is significantly reduced in CCR6^−/−^ mice (Figure 5C). It has been reported that IL-6 is a critical cytokine, which is secreted by CD11b^+^ DCs in the SED of PPs, to induce IgA production (65). Given that CCR6 is expressed on CD11b^+^ DCs of PPs (33) and that CCR6 deficiency results in reduced CD11b^+^ DCs in the SED of PPs (33), it is plausible that CCR6^−/−^ mice have reduced PP CD11b^+^ DCs and produce less IL-6, which in turn, affects IgA production.

Interestingly, the critical role for CCR6 in GC responses does not seem to be restricted to mucosal lymphoid tissues, as recent studies have shown abnormal GC responses in spleens and diminished high-affinity, antigen-specific IgG production in CCR6^−/−^ mice upon antigen challenge (66, 67). Our study, along with these previous studies, implies that in addition to CXCR5, which is known to be important for GC reactions and antibody production, CCR6 expression on B cells is also crucial for GC reactions and TD antibody production.

Memory B cells are responsible for eliciting rapid and efficient immune responses upon reexposure to the same antigen. We have demonstrated that the localization of CCR6-bearing IgA memory B cells to the SED are associated with marked expression of CCL20 in the FAE (Figure 4F) and that CCR6 signaling regulates the survival of IgA memory B cells in PPs (Figure 4E). These results demonstrate that CCR6–CCL20 axis is critical for the maintenance of IgA memory B cells by regulating their survival and recruitment to the SED. The maintenance of IgA memory B cells is particularly important for gut immunity because microfold cells (M cells) of PPs continuously receive harmful antigens/pathogens from the gut lumen. Sufficient numbers and effective function of IgA memory B cells is expected to be required for hosts to mount memory responses to antigens/pathogens. CCR6 signaling may regulate the survival of IgA memory B cells *via* the PI3K–Akt pathway (24, 68), which mediates the survival of cells, including B cells (69). In addition, we cannot exclude the involvement of CD73 in the survival of IgA memory B cells because IgA memory B cells highly express CD73 (Figure 4C), which has been shown positively associated with anti-apoptotic effects (50). Considered teleologically, the switch in CCR6 expression from a very low level on GC B cells to a very high level on IgA memory B cells (Figure 3A) may represent a critical point, not only in maintaining IgA memory B-cell pool but also in situating IgA memory B cells within the SED of PPs for efficiently mounting secondary immune responses.

Our study demonstrates that CCR6 plays an important role in TD-IgA production in PPs, in which CCR6 expression on both B cells and Th17 cells in PPs is critical for GC reactions to efficiently generate high-affinity TD-IgA. Furthermore, CCR6 expression on memory B cells is required for their survival and positioning in the SED of PPs. During the preparation of this manuscript, Reboldi et al. reported that CCR6 mediates the interaction between pre-GC B cells and DCs in the SED, which is necessary for IgA production (70). Although both studies show the important role of CCR6 in IgA production, the approaches employed, and the mechanisms proposed are quite different. First, we used WT and CCR6^−/−^ littermate mice for this study, while Reboldi et al. used bone marrow chimera and cell transfer approaches. Second, we focused on determining how CCR6 expression on pre-GC B cells and Th17 cells influences GC reactions and IgA production. In contrast, Reboldi et al. focused their study on determining how CCR6 expression on pre-GC B cells promotes their migration toward the SED to interact with DCs for IgA production.

In addition to IgA, LTi cells, a subset of ILC3s that express CCR6 and situate at cryptopatches and ILFs (71), play a critical role in maintaining intestinal homeostasis. ILC3–LTi cells are the primary source of IL-22, which stimulates IECs to produce AMPs for maintaining barrier function and mediating innate defenses (14). The significant decrease of IL-22 production in intestinal tissues of CCR6^−/−^ mice (Figures 8D,E) may be due to the small-sized ILFs containing fewer ILC3–LTi cells, suggesting the quantitatively reduced IL-22 production in CCR6^−/−^ mice. Notably, a very recent study demonstrates that RET, a receptor tyrosine kinase, is predominantly expressed on ILC3–LTi cells and the ablation of RET in ILC3s strongly reduced IL-22 production by ILC3s, suggesting that the RET signaling in ILC3–LTi cells is critical for IL-22 production (59). We found that RET expression in the crypts of CCR6^−/−^ mice is significantly reduced compared to WT mice (Figure 8D). Thus, the reduced RET levels in CCR6^−/−^ mice may also contribute to the reduced IL-22 production, suggesting an additional mechanism by which IL-22 production may be qualitatively affected in CCR6^−/−^ mice. It is plausible that CCR6 deficiency results in quantitative and qualitative decreases in IL-22 production by ILC3–LTi cells, and accordingly the diminished IL-22 impairs IEC functioning.

In addition to the maintenance of intestinal barrier, ILC3–LTi cells are important for developing tertiary lymphoid tissues (16). ILC3–LTi cells, B cells, and DCs are major components of cryptopatches and ILFs (22). Although the frequencies of RORγt-expressing cells and B cells in ILFs were comparable in CCR6^−/−^ mice and WT mice, the majority of ILFs in CCR6^−/−^ mice were small-sized ILFs, suggesting that CCR6 regulates ILF maturation (Figure 8A), consistent with previous report (72). Interestingly, we found that CCR6 deficiency alters the milieu of gut-associated lymphoid tissues (PPs and ILFs) with reduced levels of CXCL13 and CCL20 (Figures 5B and 8D). CXCL13 may be responsible for continuously recruiting B cells into the follicular structures (both PP and ILFs) at the later stage of development, as CXCL13 is the sole ligand for CXCR5 and is important for B cell chemoattraction. CCL20 may serve as chemoattractant for ILC3–LTi cells into ILFs, but may not be necessary for B cells and ILC3–LTi cells trafficking into PPs (Figures 5A and 7B). The reduced CXCL13 and CCL20 provide an explanation on the small-sized ILFs and PPs in CCR6^−/−^ mice. Of note, CCR6 deficiency seems to affect the expression of CXCL13 and CCL20. The underlying mechanism by which CCR6 signaling regulates CXCL13 and CCL20 expression requires further investigation.

Interestingly, among commensal bacteria analyzed, *Alcaligenes* and SFB were significantly increased in intestinal tissues in CCR6^−/−^ mice. Notably, both *Alicaligenes* and SFB are intestinal tissue-associated commensal bacteria; *Alcaligenes* exclusively inhabit murine PPs and SFB dominates the epithelial surface of gut-associated lymphoid structure and intestinal epithelial surface (63). These particular niches allow the bacteria to participate in the regulation of intestinal adaptive immunity (63, 73). CCR6 appears to play an important role in the control of gut pathobionts, which are known to overgrow in the absence of a strong specific adaptive immune response in the mucosa. Immune mechanisms that restrict the interactions between commensal bacteria and epithelial cells include mucus barrier, IgA secretion, and AMP production. The mucus barrier may not be critical for limiting tissue-associated commensal bacteria; however, IgA and AMPs are absolutely crucial for the containment of tissue-associated commensal bacteria. IgA, IL-17, IL-22, and AMPs have been shown to control the expansion of SFB and *Alcaligenes* (6, 74–77). The increase of SFB and *Alcaligenes* in the intestinal tissues of CCR6^−/−^ mice is likely attributable to the decrease of IgA, IL-17, IL-22, and AMP production. Using CCR6^−/−^ mice, we have demonstrated that the CCR6–CCL20 axis plays a critical role in the maintenance of intestinal symbiosis, in particular for limiting the overgrowth of tissue-associated commensal bacteria to maintain the host health. Interestingly, a recent report shows that SFB-like organisms detected in the biopsy specimens of ileo-cecal valves are more often seen and in much greater density in patients with ulcerative colitis than in control cases without bowel inflammation (78). Whether the CCR6–CCL20 axis has a role in maintaining human intestinal symbiosis warrants further investigation.

## Ethics Statement

This study was carried out in accordance with the recommendations of Institutional Guideline. The protocol was approved by the Institutional Animal Care and Utilization Committee at Academia Sinica.

## Author Contributions

Y-LL designed and performed experiments, analyzed data, and wrote the manuscript. P-PI designed and performed IFA, analyzed data, and contributed to valuable discussions. FL supervised and designed experiments, analyzed data, and wrote the manuscript.

## Conflict of Interest Statement

The authors declare that the research was conducted in the absence of any commercial or financial relationships that could be construed as a potential conflict of interest.

## References

[1] Artis D. Epithelial-cell recognition of commensal bacteria and maintenance of immune homeostasis in the gut. Nat Rev Immunol (2008) 8:411–20. 10.1038/nri2316 18469830

[2] Hooper LV Littman DR Macpherson AJ. Interactions between the microbiota and the immune system. Science (2012) 336:1268–73. 10.1126/science.1223490 22674334 PMC4420145

[3] Mazanec MB Kaetzel CS Lamm ME Fletcher D Nedrud JG. Intracellular neutralization of virus by immunoglobulin A antibodies. Proc Natl Acad Sci U S A (1992) 89:6901–5. 10.1073/pnas.89.15.6901 1323121 PMC49612

[4] Lycke N Eriksen L Holmgren J. Protection against cholera toxin after oral immunization is thymus-dependent and associated with intestinal production of neutralizing IgA antitoxin. Scand J Immunol (1987) 25:413–9. 10.1111/j.1365-3083.1987.tb02208.x 3576135

[5] Cash HL Whitham CV Behrendt CL Hooper LV. Symbiotic bacteria direct expression of an intestinal bactericidal lectin. Science (2006) 313:1126–30. 10.1126/science.1127119 16931762 PMC2716667

[6] Vaishnava S Yamamoto M Severson KM Ruhn KA Yu X Koren O The antibacterial lectin RegIIIgamma promotes the spatial segregation of microbiota and host in the intestine. Science (2011) 334:255–8. 10.1126/science.1209791 21998396 PMC3321924

[7] Craig SW Cebra JJ Peyer’s patches: an enriched source of precursors for IgA-producing immunocytes in the rabbit. J Exp Med (1971) 134:188–200. 10.1084/jem.134.1.188 4934147 PMC2139023

[8] Guy-Grand D Griscelli C Vassalli P. Peyer’s patches, gut IgA plasma cells and thymic function: study in nude mice bearing thymic grafts. J Immunol (1975) 115:361–4. 807633

[9] Hornquist CE Ekman L Grdic KD Schon K Lycke NY. Paradoxical IgA immunity in CD4-deficient mice. Lack of cholera toxin-specific protective immunity despite normal gut mucosal IgA differentiation. J Immunol (1995) 155:2877–87. 7673704

[10] Fagarasan S Kawamoto S Kanagawa O Suzuki K. Adaptive immune regulation in the gut: T cell-dependent and T cell-independent IgA synthesis. Annu Rev Immunol (2010) 28:243–73. 10.1146/annurev-immunol-030409-101314 20192805

[11] Christa L Carnot F Simon MT Levavasseur F Stinnakre MG Lasserre C HIP/PAP is an adhesive protein expressed in hepatocarcinoma, normal Paneth, and pancreatic cells. Am J Physiol (1996) 271:G993–1002. 8997243 10.1152/ajpgi.1996.271.6.G993

[12] O’Neil DA Porter EM Elewaut D Anderson GM Eckmann L Ganz T Expression and regulation of the human beta-defensins hBD-1 and hBD-2 in intestinal epithelium. J Immunol (1999) 163:6718–24. 10586069

[13] Selsted ME Ouellette AJ. Mammalian defensins in the antimicrobial immune response. Nat Immunol (2005) 6:551–7. 10.1038/ni1206 15908936

[14] Sanos SL Bui VL Mortha A Oberle K Heners C Johner C RORgammat and commensal microflora are required for the differentiation of mucosal interleukin 22-producing NKp46+ cells. Nat Immunol (2009) 10:83–91. 10.1038/ni.1684 19029903 PMC4217274

[15] Sun Z Unutmaz D Zou YR Sunshine MJ Pierani A Brenner-Morton S Requirement for RORgamma in thymocyte survival and lymphoid organ development. Science (2000) 288:2369–73. 10.1126/science.288.5475.2369 10875923

[16] Eberl G Marmon S Sunshine MJ Rennert PD Choi Y Littman DR. An essential function for the nuclear receptor RORgamma(t) in the generation of fetal lymphoid tissue inducer cells. Nat Immunol (2004) 5:64–73. 10.1038/ni1022 14691482

[17] Satoh-Takayama N Vosshenrich CA Lesjean-Pottier S Sawa S Lochner M Rattis F Microbial flora drives interleukin 22 production in intestinal NKp46+ cells that provide innate mucosal immune defense. Immunity (2008) 29:958–70. 10.1016/j.immuni.2008.11.001 19084435

[18] Klose CS Kiss EA Schwierzeck V Ebert K Hoyler T d’Hargues Y A T-bet gradient controls the fate and function of CCR6-RORgammat+ innate lymphoid cells. Nature (2013) 494:261–5. 10.1038/nature11813 23334414

[19] Takatori H Kanno Y Watford WT Tato CM Weiss G Ivanov II Lymphoid tissue inducer-like cells are an innate source of IL-17 and IL-22. J Exp Med (2009) 206:35–41. 10.1084/jem.20072713 19114665 PMC2626689

[20] Yoshida H Honda K Shinkura R Adachi S Nishikawa S Maki K IL-7 receptor alpha+ CD3(-) cells in the embryonic intestine induces the organizing center of Peyer’s patches. Int Immunol (1999) 11:643–55. 10.1093/intimm/11.5.643 10330270

[21] Honda K Nakano H Yoshida H Nishikawa S Rennert P Ikuta K Molecular basis for hematopoietic/mesenchymal interaction during initiation of Peyer’s patch organogenesis. J Exp Med (2001) 193:621–30. 10.1084/jem.193.5.621 11238592 PMC2193398

[22] Hamada H Hiroi T Nishiyama Y Takahashi H Masunaga Y Hachimura S Identification of multiple isolated lymphoid follicles on the antimesenteric wall of the mouse small intestine. J Immunol (2002) 168:57–64. 10.4049/jimmunol.168.1.57 11751946

[23] Liao F Shirakawa AK Foley JF Rabin RL Farber JM Human B cells become highly responsive to macrophage-inflammatory protein-3/CC chemokine ligand-20 after cellular activation without changes in CCR6 expression or ligand binding. J Immunol (2002) 168:4871–80. 10.4049/jimmunol.168.10.4871 11994436

[24] Shirakawa AK Liao F Zhang HH Hedrick MN Singh SP Wu D Pathway-selective suppression of chemokine receptor signaling in B cells by LPS through downregulation of PLC-beta2. Cell Mol Immunol (2010) 7:428–39. 10.1038/cmi.2010.46 20871625 PMC3343318

[25] Liao F Rabin RL Smith CS Sharma G Nutman TB Farber JM CC-chemokine receptor 6 is expressed on diverse memory subsets of T cells and determines responsiveness to macrophage inflammatory protein 3 alpha. J Immunol (1999) 162:186–94. 9886385

[26] Singh SP Zhang HH Foley JF Hedrick MN Farber JM. Human T cells that are able to produce IL-17 express the chemokine receptor CCR6. J Immunol (2008) 180:214–21. 10.4049/jimmunol.180.1.214 18097022

[27] Yamazaki T Yang XO Chung Y Fukunaga A Nurieva R Pappu B CCR6 regulates the migration of inflammatory and regulatory T cells. J Immunol (2008) 181:8391–401. 10.4049/jimmunol.181.12.8391 19050256 PMC2752441

[28] Greaves DR Wang W Dairaghi DJ Dieu MC Saint-Vis B Franz-Bacon K CCR6, a CC chemokine receptor that interacts with macrophage inflammatory protein 3alpha and is highly expressed in human dendritic cells. J Exp Med (1997) 186:837–44. 10.1084/jem.186.6.837 9294138 PMC2199049

[29] Wan W Lim JK Lionakis MS Rivollier A McDermott DH Kelsall BL Genetic deletion of chemokine receptor Ccr6 decreases atherogenesis in ApoE-deficient mice. Circ Res (2011) 109:374–81. 10.1161/CIRCRESAHA.111.242578 21680896 PMC3151346

[30] Baba M Imai T Nishimura M Kakizaki M Takagi S Hieshima K Identification of CCR6, the specific receptor for a novel lymphocyte-directed CC chemokine LARC. J Biol Chem (1997) 272:14893–8. 10.1074/jbc.272.23.14893 9169459

[31] Liao F Alderson R Su J Ullrich SJ Kreider BL Farber JM. STRL22 is a receptor for the CC chemokine MIP-3alpha. Biochem Biophys Res Commun (1997) 236:212–7. 10.1006/bbrc.1997.6936 9223454

[32] Cook DN Prosser DM Forster R Zhang J Kuklin NA Abbondanzo SJ CCR6 mediates dendritic cell localization, lymphocyte homeostasis, and immune responses in mucosal tissue. Immunity (2000) 12:495–503. 10.1016/S1074-7613(00)80201-0 10843382

[33] Iwasaki A Kelsall BL. Localization of distinct Peyer’s patch dendritic cell subsets and their recruitment by chemokines macrophage inflammatory protein (MIP)-3alpha, MIP-3beta, and secondary lymphoid organ chemokine. J Exp Med (2000) 191:1381–94. 10.1084/jem.191.8.1381 10770804 PMC2193144

[34] Williams IR. CCR6 and CCL20: partners in intestinal immunity and lymphorganogenesis. Ann N Y Acad Sci (2006) 1072:52–61. 10.1196/annals.1326.036 17057190

[35] Salazar-Gonzalez RM Niess JH Zammit DJ Ravindran R Srinivasan A Maxwell JR CCR6-mediated dendritic cell activation of pathogen-specific T cells in Peyer’s patches. Immunity (2006) 24:623–32. 10.1016/j.immuni.2006.02.015 16713979 PMC2855652

[36] Kallal LE Schaller MA Lindell DM Lira SA Lukacs NW. CCL20/CCR6 blockade enhances immunity to RSV by impairing recruitment of DC. Eur J Immunol (2010) 40:1042–52. 10.1002/eji.200939778 20101616 PMC2952176

[37] Westphal S Lugering A von Wedel J von Eiff C Maaser C Spahn T Resistance of chemokine receptor 6-deficient mice to *Yersinia enterocolitica* infection: evidence of defective M-cell formation in vivo. Am J Pathol (2008) 172:671–80. 10.2353/ajpath.2008.070393 18258848 PMC2258262

[38] Kaser A Ludwiczek O Holzmann S Moschen AR Weiss G Enrich B Increased expression of CCL20 in human inflammatory bowel disease. J Clin Immunol (2004) 24:74–85. 10.1023/B:JOCI.0000018066.46279.6b 14997037

[39] Barrett JC Hansoul S Nicolae DL Cho JH Duerr RH Rioux JD Genome-wide association defines more than 30 distinct susceptibility loci for Crohn’s disease. Nat Genet (2008) 40:955–62. 10.1038/ng.175 18587394 PMC2574810

[40] Hedrick MN Lonsdorf AS Shirakawa A-K Lee C-CR Liao F Singh SP CCR6 is required for IL-23–induced psoriasis-like inflammation in mice. J Clin Invest (2009) 119:2317–29. 10.1172/JCI37378 19662682 PMC2719919

[41] Reissig S Hackenbruch C Hovelmeyer N Isolation of T cells from the gut. Methods Mol Biol (2014) 1193:21–5. 10.1007/978-1-4939-1212-4_3 25150993

[42] Kawamoto S Tran TH Maruya M Suzuki K Doi Y Tsutsui Y The inhibitory receptor PD-1 regulates IgA selection and bacterial composition in the gut. Science (2012) 336:485–9. 10.1126/science.1217718 22539724

[43] Barman M Unold D Shifley K Amir E Hung K Bos N Enteric salmonellosis disrupts the microbial ecology of the murine gastrointestinal tract. Infect Immun (2008) 76:907–15. 10.1128/IAI.01432-07 18160481 PMC2258829

[44] Nakano M Niwa M Nishimura N. Development of a PCR-based method for monitoring the status of *Alcaligenes* species in the agricultural environment. Biocontrol Sci (2014) 19:23–31. 10.4265/bio.19.23 24670615

[45] Varona R Villares R Carramolino L Goya F Zaballos A Gutieerrez J CCR6-deficient mice have impaired leukocyte homeostasis and altered contact hypersensitivity and delayed-type hypersensitivity responses. J Clin Invest (2001) 107:R37–45. 10.1172/JCI11297 11254677 PMC208945

[46] Macpherson AJ McCoy KD Johansen FE Brandtzaeg P. The immune geography of IgA induction and function. Mucosal Immunol (2008) 1:11–22. 10.1038/mi.2007.6 19079156

[47] Kitano M Moriyama S Ando Y Hikida M Mori Y Kurosaki T Bcl6 protein expression shapes pre-germinal center B cell dynamics and follicular helper T cell heterogeneity. Immunity (2011) 34:961–72. 10.1016/j.immuni.2011.03.025 21636294

[48] Basso K Schneider C Shen Q Holmes AB Setty M Leslie C BCL6 positively regulates AID and germinal center gene expression via repression of miR-155. J Exp Med (2012) 209:2455–65. 10.1084/jem.20121387 23166356 PMC3526356

[49] Tomayko MM Steinel NC Anderson SM Shlomchik MJ. Cutting edge: hierarchy of maturity of murine memory B cell subsets. J Immunol (2010) 185:7146–50. 10.4049/jimmunol.1002163 21078902 PMC3133669

[50] Serra S Horenstein AL Vaisitti T Brusa D Rossi D Laurenti L CD73-generated extracellular adenosine in chronic lymphocytic leukemia creates local conditions counteracting drug-induced cell death. Blood (2011) 118:6141–52. 10.1182/blood-2011-08-374728 21998208 PMC3342854

[51] Okada T Ngo VN Ekland EH Forster R Lipp M Littman DR Chemokine requirements for B cell entry to lymph nodes and Peyer’s patches. J Exp Med (2002) 196:65–75. 10.1084/jem.20020201 12093871 PMC2194009

[52] Mitsdoerffer M Lee Y Jager A Kim HJ Korn T Kolls JK Proinflammatory T helper type 17 cells are effective B-cell helpers. Proc Natl Acad Sci U S A (2010) 107:14292–7. 10.1073/pnas.1009234107 20660725 PMC2922571

[53] Hirota K Turner JE Villa M Duarte JH Demengeot J Steinmetz OM Plasticity of Th17 cells in Peyer’s patches is responsible for the induction of T cell-dependent IgA responses. Nat Immunol (2013) 14:372–9. 10.1038/ni.2552 23475182 PMC3672955

[54] Sawa S Cherrier M Lochner M Satoh-Takayama N Fehling HJ Langa F Lineage relationship analysis of RORgammat+ innate lymphoid cells. Science (2010) 330:665–9. 10.1126/science.1194597 20929731

[55] Bando JK Colonna M. Innate lymphoid cell function in the context of adaptive immunity. Nat Immunol (2016) 17:783–9. 10.1038/ni.3484 27328008 PMC5156404

[56] Hepworth MR Fung TC Masur SH Kelsen JR McConnell FM Dubrot J Immune tolerance. Group 3 innate lymphoid cells mediate intestinal selection of commensal bacteria-specific CD4(+) T cells. Science (2015) 348:1031–5. 10.1126/science.aaa4812 25908663 PMC4449822

[57] Wang C Kang SG Lee J Sun Z Kim CH. The roles of CCR6 in migration of Th17 cells and regulation of effector T-cell balance in the gut. Mucosal Immunol (2009) 2:173–83. 10.1038/mi.2008.84 19129757 PMC2709747

[58] Lindemans CA Calafiore M Mertelsmann AM O’Connor MH Dudakov JA Jenq RR Interleukin-22 promotes intestinal-stem-cell-mediated epithelial regeneration. Nature (2015) 528:560–4. 10.1038/nature16460 26649819 PMC4720437

[59] Ibiza S Garcia-Cassani B Ribeiro H Carvalho T Almeida L Marques R Glial-cell-derived neuroregulators control type 3 innate lymphoid cells and gut defence. Nature (2016) 535:440–3. 10.1038/nature18644 27409807 PMC4962913

[60] Derebe MG Zlatkov CM Gattu S Ruhn KA Vaishnava S Diehl GE Serum amyloid A is a retinol binding protein that transports retinol during bacterial infection. Elife (2014) 3:e03206. 10.7554/eLife.03206 25073702 PMC4129439

[61] Pabst O. New concepts in the generation and functions of IgA. Nat Rev Immunol (2012) 12:821–32. 10.1038/nri3322 23103985

[62] Artis D Spits H. The biology of innate lymphoid cells. Nature (2015) 517:293–301. 10.1038/nature14189 25592534

[63] Obata T Goto Y Kunisawa J Sato S Sakamoto M Setoyama H Indigenous opportunistic bacteria inhabit mammalian gut-associated lymphoid tissues and share a mucosal antibody-mediated symbiosis. Proc Natl Acad Sci U S A (2010) 107:7419–24. 10.1073/pnas.1001061107 20360558 PMC2867693

[64] Vinuesa CG Linterman MA Yu D MacLennan IC. Follicular helper T cells. Annu Rev Immunol (2016) 34:335–68. 10.1146/annurev-immunol-041015-055605 26907215

[65] Sato A Hashiguchi M Toda E Iwasaki A Hachimura S Kaminogawa S. CD11b+ Peyer’s patch dendritic cells secrete IL-6 and induce IgA secretion from naive B cells. J Immunol (2003) 171:3684–90. 10.4049/jimmunol.171.7.3684 14500666

[66] Wiede F Fromm PD Comerford I Kara E Bannan J Schuh W CCR6 is transiently upregulated on B cells after activation and modulates the germinal center reaction in the mouse. Immunol Cell Biol (2013) 91:335–9. 10.1038/icb.2013.14 23588497

[67] Reimer D Lee AY Bannan J Fromm P Kara EE Comerford I Early CCR6 expression on B cells modulates germinal centre kinetics and efficient antibody responses. Immunol Cell Biol (2017) 95:33–41. 10.1038/icb.2016.68 27465674

[68] Lin SL Chien CW Han CL Chen ES Kao SH Chen YJ Temporal proteomics profiling of lipid rafts in CCR6-activated T cells reveals the integration of actin cytoskeleton dynamics. J Proteome Res (2010) 9:283–97. 10.1021/pr9006156 19928914

[69] Pogue SL Kurosaki T Bolen J Herbst R. B cell antigen receptor-induced activation of Akt promotes B cell survival and is dependent on Syk kinase. J Immunol (2000) 165:1300–6. 10.4049/jimmunol.165.3.1300 10903730

[70] Reboldi A Arnon TI Rodda LB Atakilit A Sheppard D Cyster JG. IgA production requires B cell interaction with subepithelial dendritic cells in Peyer’s patches. Science (2016) 352:aaf4822. 10.1126/science.aaf4822 27174992 PMC4890166

[71] Lugering A Ross M Sieker M Heidemann J Williams IR Domschke W CCR6 identifies lymphoid tissue inducer cells within cryptopatches. Clin Exp Immunol (2010) 160:440–9. 10.1111/j.1365-2249.2010.04103.x 20148914 PMC2883115

[72] McDonald KG McDonough JS Wang C Kucharzik T Williams IR Newberry RD. CC chemokine receptor 6 expression by B lymphocytes is essential for the development of isolated lymphoid follicles. Am J Pathol (2007) 170:1229–40. 10.2353/ajpath.2007.060817 17392163 PMC1829457

[73] Lecuyer E Rakotobe S Lengline-Garnier H Lebreton C Picard M Juste C Segmented filamentous bacterium uses secondary and tertiary lymphoid tissues to induce gut IgA and specific T helper 17 cell responses. Immunity (2014) 40:608–20. 10.1016/j.immuni.2014.03.009 24745335

[74] Suzuki K Meek B Doi Y Muramatsu M Chiba T Honjo T Aberrant expansion of segmented filamentous bacteria in IgA-deficient gut. Proc Natl Acad Sci U S A (2004) 101:1981–6. 10.1073/pnas.0307317101 14766966 PMC357038

[75] Kumar P Monin L Castillo P Elsegeiny W Horne W Eddens T Intestinal interleukin-17 receptor signaling mediates reciprocal control of the gut microbiota and autoimmune inflammation. Immunity (2016) 44:659–71. 10.1016/j.immuni.2016.02.007 26982366 PMC4794750

[76] Sonnenberg GF Monticelli LA Alenghat T Fung TC Hutnick NA Kunisawa J Innate lymphoid cells promote anatomical containment of lymphoid-resident commensal bacteria. Science (2012) 336:1321–5. 10.1126/science.1222551 22674331 PMC3659421

[77] Goto Y Obata T Kunisawa J Sato S Ivanov II Lamichhane A Innate lymphoid cells regulate intestinal epithelial cell glycosylation. Science (2014) 345:1254009. 10.1126/science.1254009 25214634 PMC4774895

[78] Caselli M Tosini D Gafa R Gasbarrini A Lanza G Segmented filamentous bacteria-like organisms in histological slides of ileo-cecal valves in patients with ulcerative colitis. Am J Gastroenterol (2013) 108:860–1. 10.1038/ajg.2013.61 23644973

