# Supplementary material for: CCR6 Deficiency Impairs IgA Production and Dysregulates Antimicrobial Peptide Production, Altering the Intestinal Flora
Source: Front Immunol. 2017 Jul 11;8:805. doi: 10.3389/fimmu.2017.00805 (PMC5504188; doi:10.3389/fimmu.2017.00805)
Supplement: Supplementary file 1 [file Data_Sheet_1.pdf]

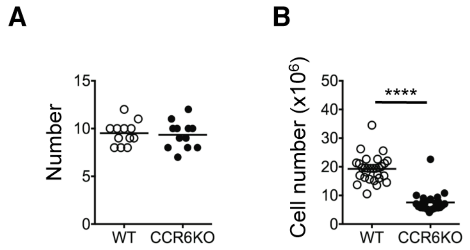

Supplementary Figure S1. CCR6$^{-/-}$ mice have small size of PPs. Mice were sacrificed and PPs were isolated from small intestines. **A.** Number of PPs in one mouse is shown. **B.** Total lymphocyte number of PPs in one mouse is shown. Each symbol represents one mouse. Data are a compilation of three **(A)** or six **(B)** independent experiments. ****, *P* < 0.0001.

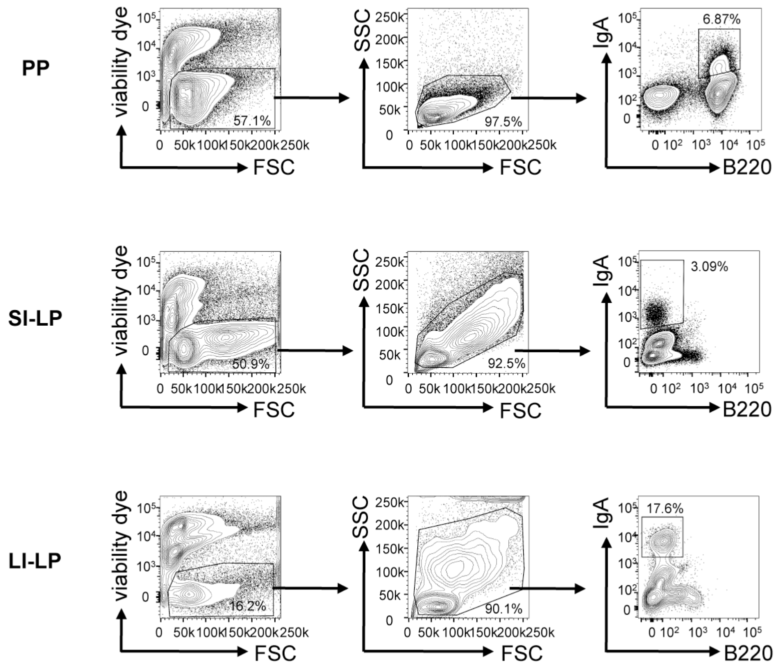

Supplementary Figure S2. Gating strategy to detect IgA-bearing cells in the intestinal tissues. Dead cells were excluded using a viability dye. Leukocytes were gated using a FSC vs. SSC plot. IgA-bearing B cells (IgA^+B220^+ in PP) and IgA-bearing plasma cells (IgA^+B220^- in SI-LP and LI-LP) were further identified by surface staining of B220 and IgA.

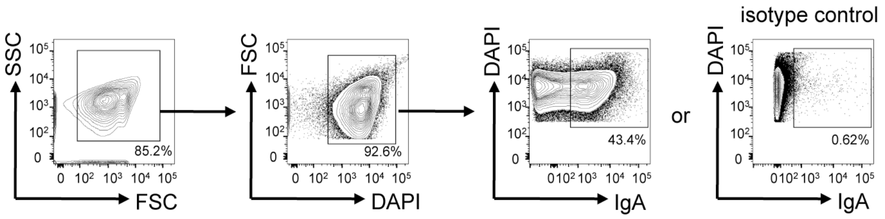

Supplementary Figure S3. Gating strategy to detect IgA-coated fecal bacteria. Bacteria were gated using a FSC vs. SSC plot and DAPI positive events were gated for further analysis. Bacteria coated with mouse IgA were identified by IgA-positive events. IgA isotype control (far right panel) was also included for each bacteria sample.

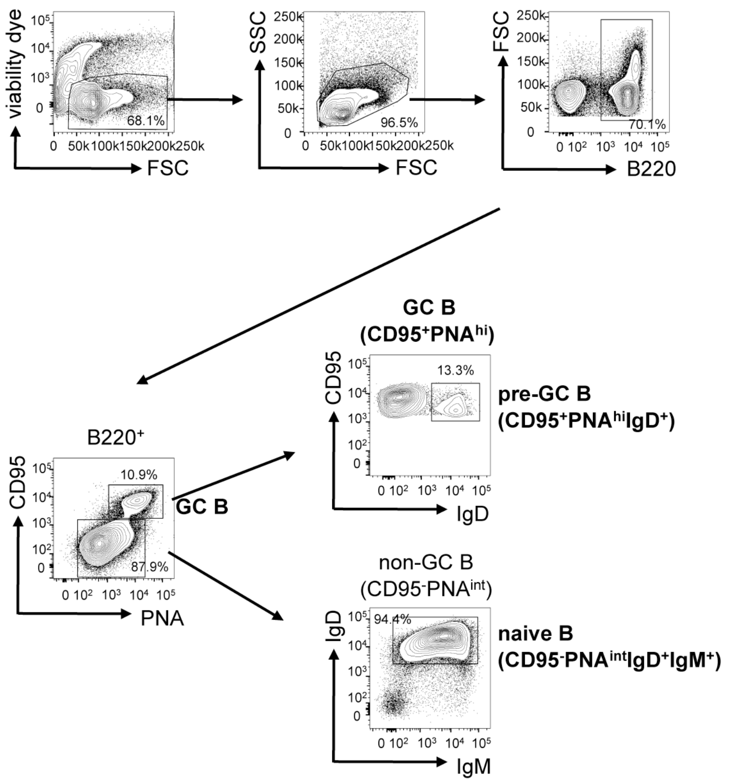

Supplementary Figure S4. Gating strategy to detect naive B, pre-GC B and GC B cells by staining cells with antibodies to B220, IgD, IgM, CD95 and PNA. Dead cells were excluded using a viability dye. PP lymphocytes were gated using a FSC vs. SSC plot. B220$^+$ cells were identified as PP B cells. Within B220$^+$ population, cells were further identified as GC B cells (CD95$^+$PNA^{hi}) and non-GCB cells (CD95$^-$PNA^{int}). Pre-GC B cells were further identified as IgD$^+$ cells within GC B cells. Naive B cells were further identified as IgD$^+$IgM$^+$ cells within non GC-B cells.

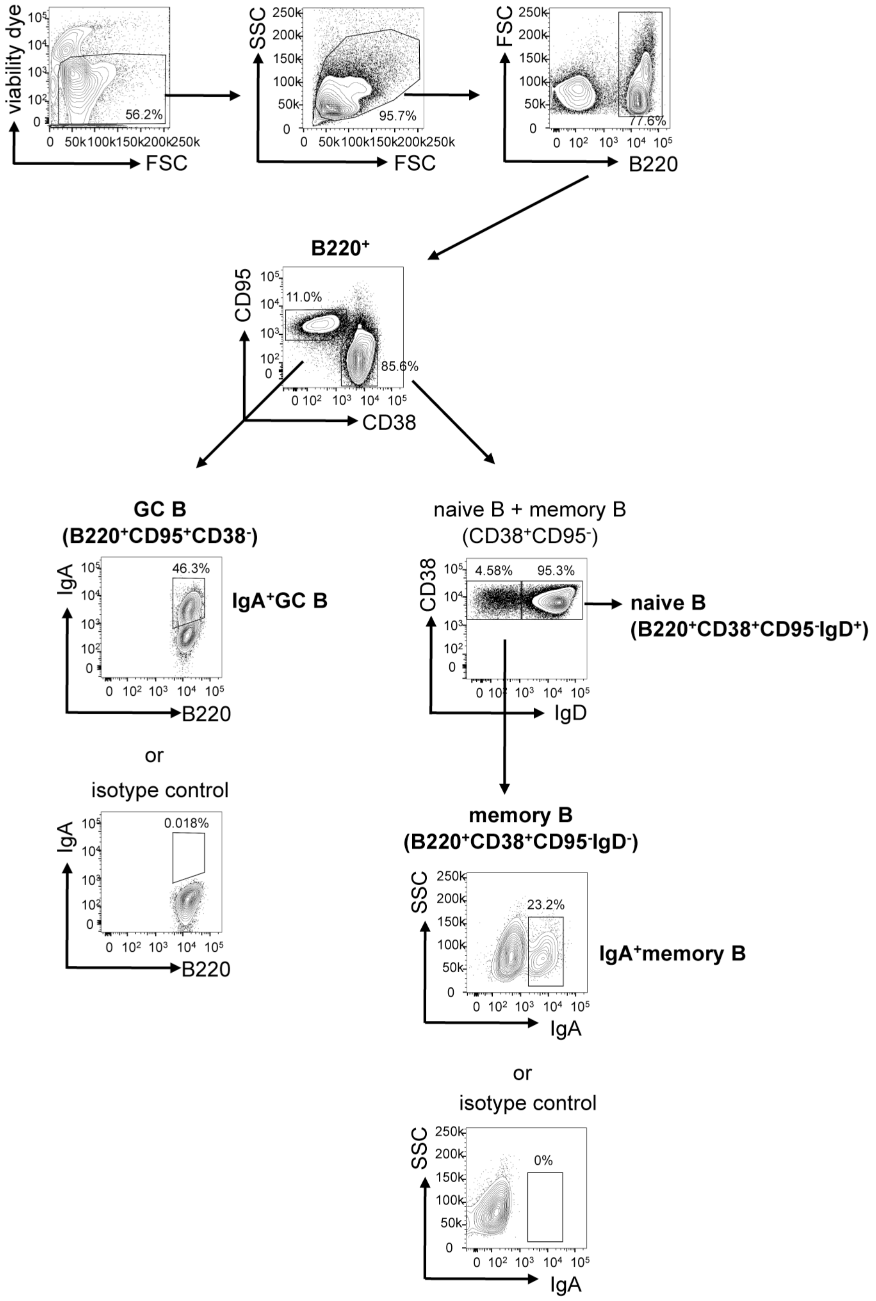

Supplementary Figure S5. Gating strategy to detect naive B, GC B, IgA-bearing GC B and IgA-bearing memory B cells by staining cells with antibodies to B220, IgD, CD95, CD38 and surface IgA. Dead cells were excluded using a viability dye. PP lymphocytes were gated using a FSC vs. SSC plot. B220$^+$ cells were identified as PP B cells. PP B cells were further identified as GC B cells (CD95$^+$CD38$^-$) or naive and memory B cells (CD95$^-$CD38$^+$). IgA$^+$ GC B cells were further identified within GC B cells. Naive B cells were further identified as IgD$^+$ cells within the CD95$^-$CD38$^+$ population and memory B cells were further identified as IgD$^-$ cells within the CD95$^-$ CD38$^+$ population. IgA$^+$ memory B cells were identified as surface IgA$^+$ within the memory B cell population.

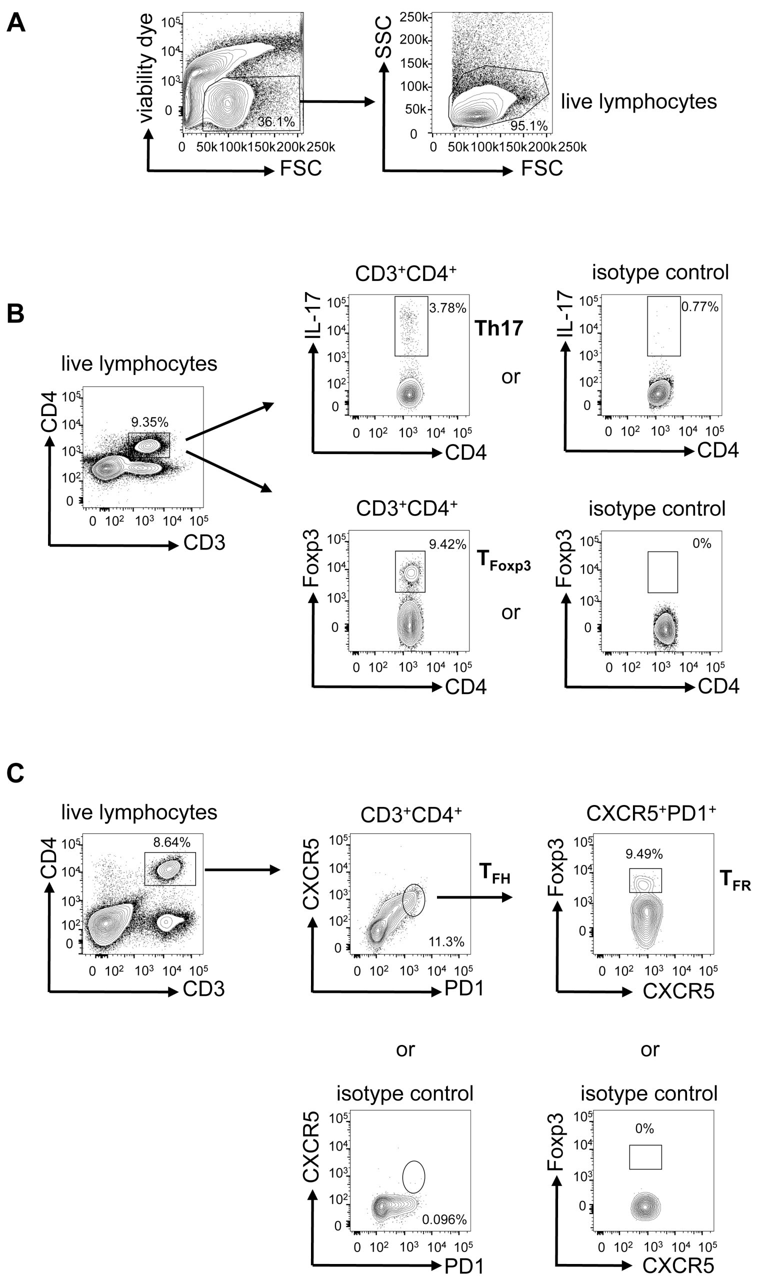

Supplementary Figure S6. Gating strategy to detect PP T cell subsets. A. Dead cells were excluded using a viability dye. PP lymphocytes were gated using a FSC vs. SSC plot. **B**. $CD4^+$ T helper cells were gated on $CD3^+CD4^+$ cells within the lymphocyte population. Th17 cells were further identified as $CD3^+CD4^+IL\text{-}17^+$ T cells and the isotype control for anti-IL-17 was also included. T_{Foxp3} cells were further identifies as $CD3^+CD4^+Foxp3^+$ cells and the isotype control for anti-Foxp3 was also included. **C**. T_{FH} cells were identified as $CD3^+CD4^+CXCR5^+PD1^+$ cells, and T_{FR} cells were further gated on $Foxp3^+$ cells within the T_{FH} population. The isotype controls for anti-CXCR5 and for anti-Foxp3 were also included.

Supplement Table S1. Primer sequences and the Taqman probes used in this study.

(A) Primer sequences used for qPCR

murine gene	forward	reverse
Reg3β	ATGGCTCCTACTGCTATGCC	GTGTCCTCCAGGCCTCTTT
Reg3γ	ATGGCTCCTATTGCTATGCC	GATGTCCTGAGGGCCTCTT
SAA1	CATTTGTTCACGAGGCTTTCC	GTTTTTCCAGTTAGCTTCCTTCATGT
SAA2	TGTGTATCCCACAAGGTTTCAGA	TTATTACCCTCTCCTCCTCAAGCA
CCL20	TACGACTGTTGCCTCTCGTACATAC	TCTGTGCAGTGATGTGCAGGTGAA
CXCL13	TTACGCCCCCTGGGAATGGCT	GGGTATGCAACGGAGCTTGAGCA
CD79α	GAACCACAGGGGCTTGTACT	GGTTCTTGGTACCTTCCCCC
LTα	TCCACTCCCTCAGAAGCACT	AGAGAAGCCATGTCGGAGAA
LTβ	TACACCAGATCCAGGGGTTC	ACTCATCCAAGCGCCTATGA
TGF-β1	AAGGCTCGCCAGTCCCCCAA	TAGATGGCGTTGTTGCGGTCCAC
IL-21	AGGGCCAGATCGCCTCCTGA	GCATGCTCACAGTGCCCCTTTACA
BAFF	AGGCTGGAAGAAGGAGATGAG	CAGAGAAGACGAGGGAAGGG
APRIL	GGGGAAGGAGTGTCAGAGTG	GCAGGGAGGGTGGGAATAC
GAPDH	TACTTGGCAGGTTTCTCCAG	GTCGTGGAGTCTACTGGTGT

(B) Gene expression analyzed with Taqman probes

murine gene	Taqman probe
IL-22	Mm04203745_mH
IL-17A	Mm00439618_m1
RORc	Mm01261022_m1
IL-10	Mm00439614_m1
IL-6	Mm00446190_ml
GM-CSF	Mm01290062 _m1
GAPDH	Mm99999915_g1
